# Correction: Effect of exercise preconditioning on myocardial content of Sphingosine1-phosphate and its mechanism in rats after exhaustive exercise

**DOI:** 10.1371/journal.pone.0347503

**Published:** 2026-04-16

**Authors:** Xinnuan Wei, Weiyuan Yang, Junxiang Zhou, Luoyuan Cao, Wenxing Jiang

There are errors in the author affiliations. The correct affiliations are as follows:

Xinnuan Wei^1,2^, Weiyuan Yang^1,2^, Junxiang Zhou^1,2^, Luoyuan Cao^3^, Wenxing Jiang^1,2^

**1** Ningde Clinical Medical College of Fujian Medical University, Ningde, Fujian, People’s Republic of China, **2** Department of Rehabilitation Medicine, Ningde Municipal Hospital of Ningde Normal University, Ningde, Fujian, People’s Republic of China, **3** Department of Central Laboratory, Ningde Municipal Hospital of Ningde Normal University, Ningde, Fujian, People’s Republic of China
