# Supplementary figures and images for: Effect of exercise preconditioning on myocardial content of Sphingosine1-phosphate and its mechanism in rats after exhaustive exercise
Source: PLoS One. 2026 Jan 7;21(1):e0340313. doi: 10.1371/journal.pone.0340313 (PMC12779141; doi:10.1371/journal.pone.0340313)

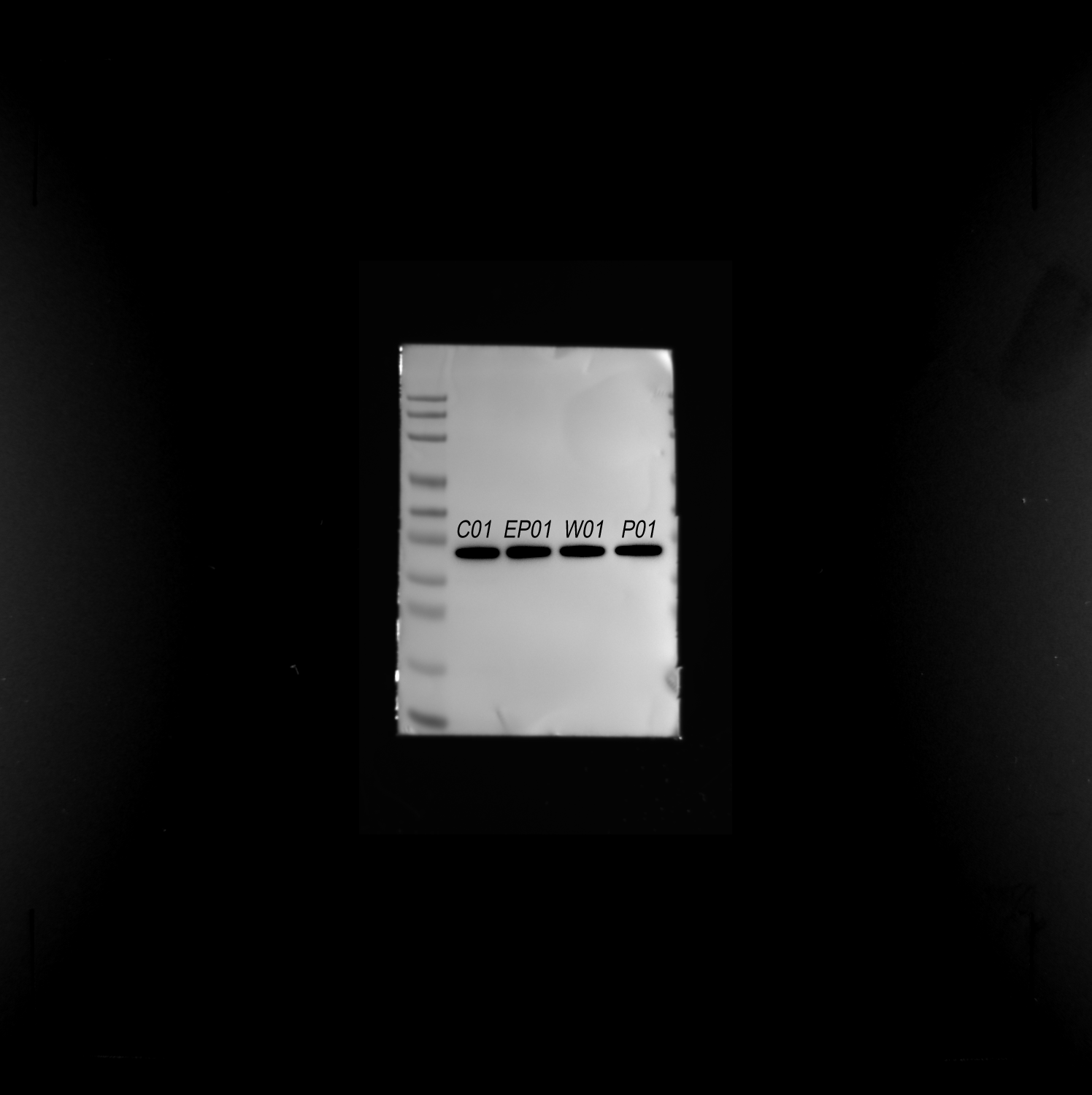

Supplement: S1 File — Contains the complete set of original, uncropped, and unprocessed Western blot images from which the data presented in this study were derived. The lanes containing the molecular weight markers are present. The corresponding molecular weight calibration reference for these markers is provided in S2 File. (ZIP) [file pone.0340313.s001.zip › WB-raw-images/Membrane1_GAPDH_annotated_C01-EP01-W01-P01_original.tif]

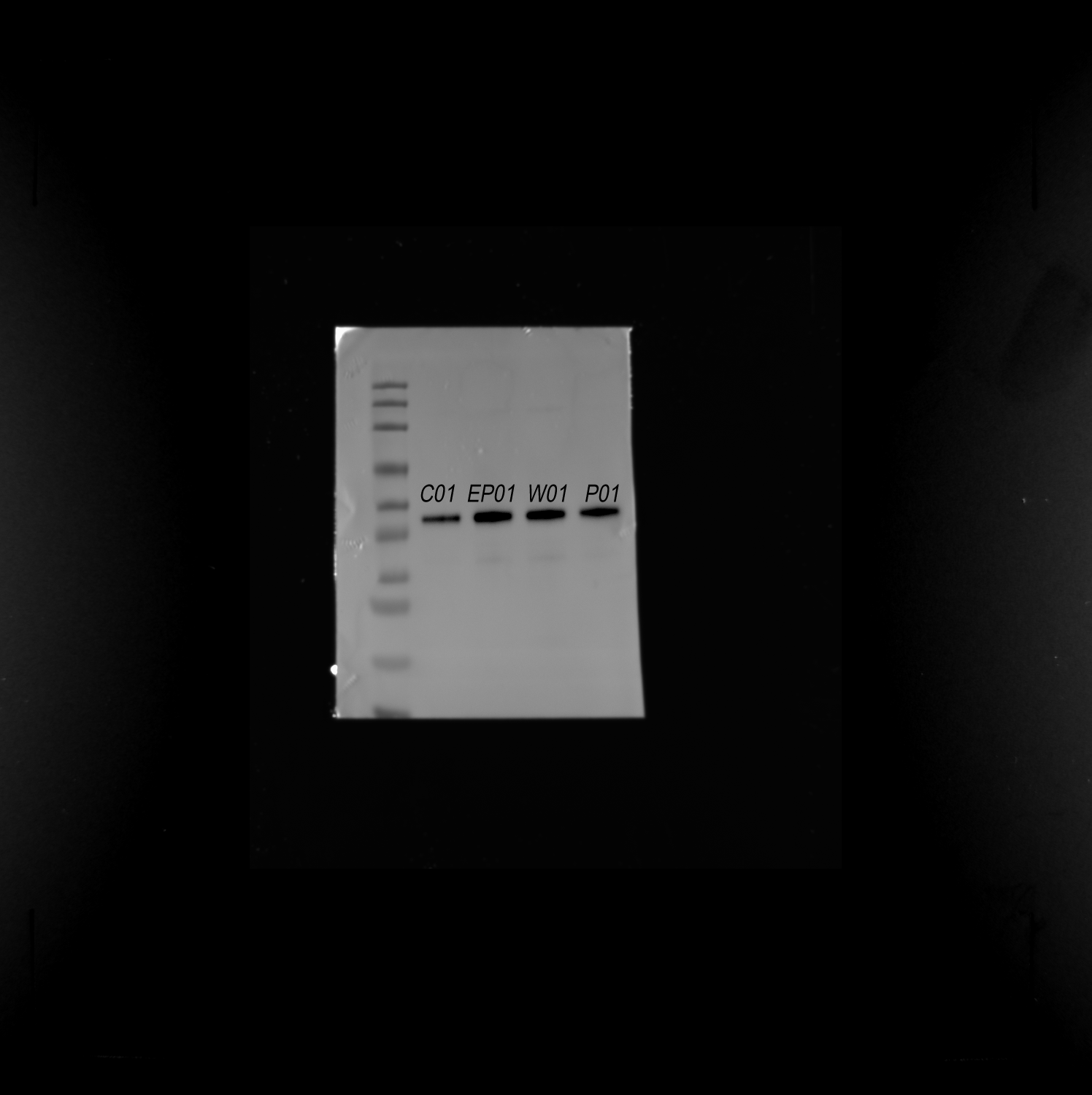

Supplement: S1 File — Contains the complete set of original, uncropped, and unprocessed Western blot images from which the data presented in this study were derived. The lanes containing the molecular weight markers are present. The corresponding molecular weight calibration reference for these markers is provided in S2 File. (ZIP) [file pone.0340313.s001.zip › WB-raw-images/Membrane1_PMEK_annotated_C01-EP01-W01-P01_original.tif]

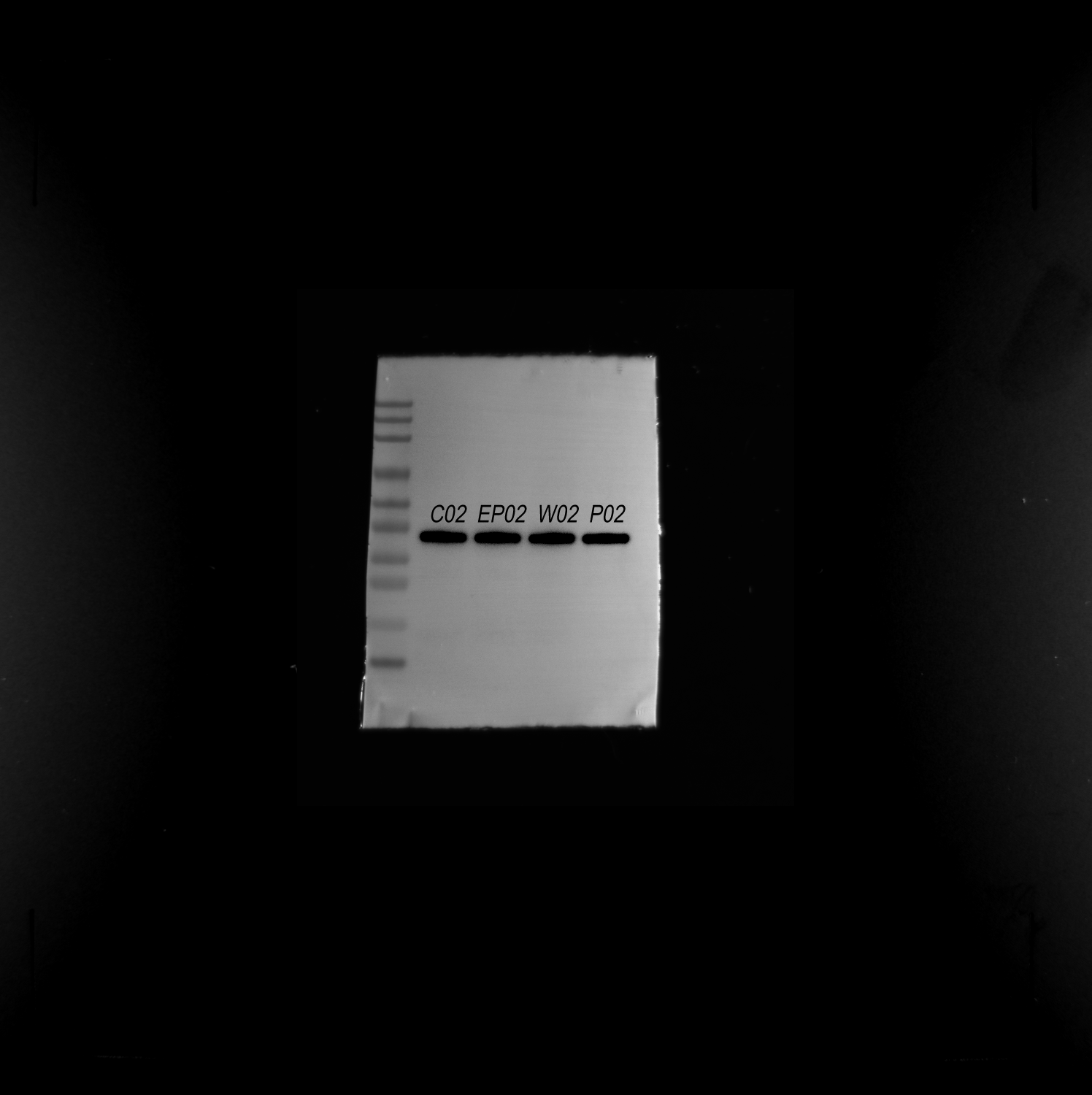

Supplement: S1 File — Contains the complete set of original, uncropped, and unprocessed Western blot images from which the data presented in this study were derived. The lanes containing the molecular weight markers are present. The corresponding molecular weight calibration reference for these markers is provided in S2 File. (ZIP) [file pone.0340313.s001.zip › WB-raw-images/Membrane2_GAPDH_annotated_C02-EP02-W02-P02_original.tif]

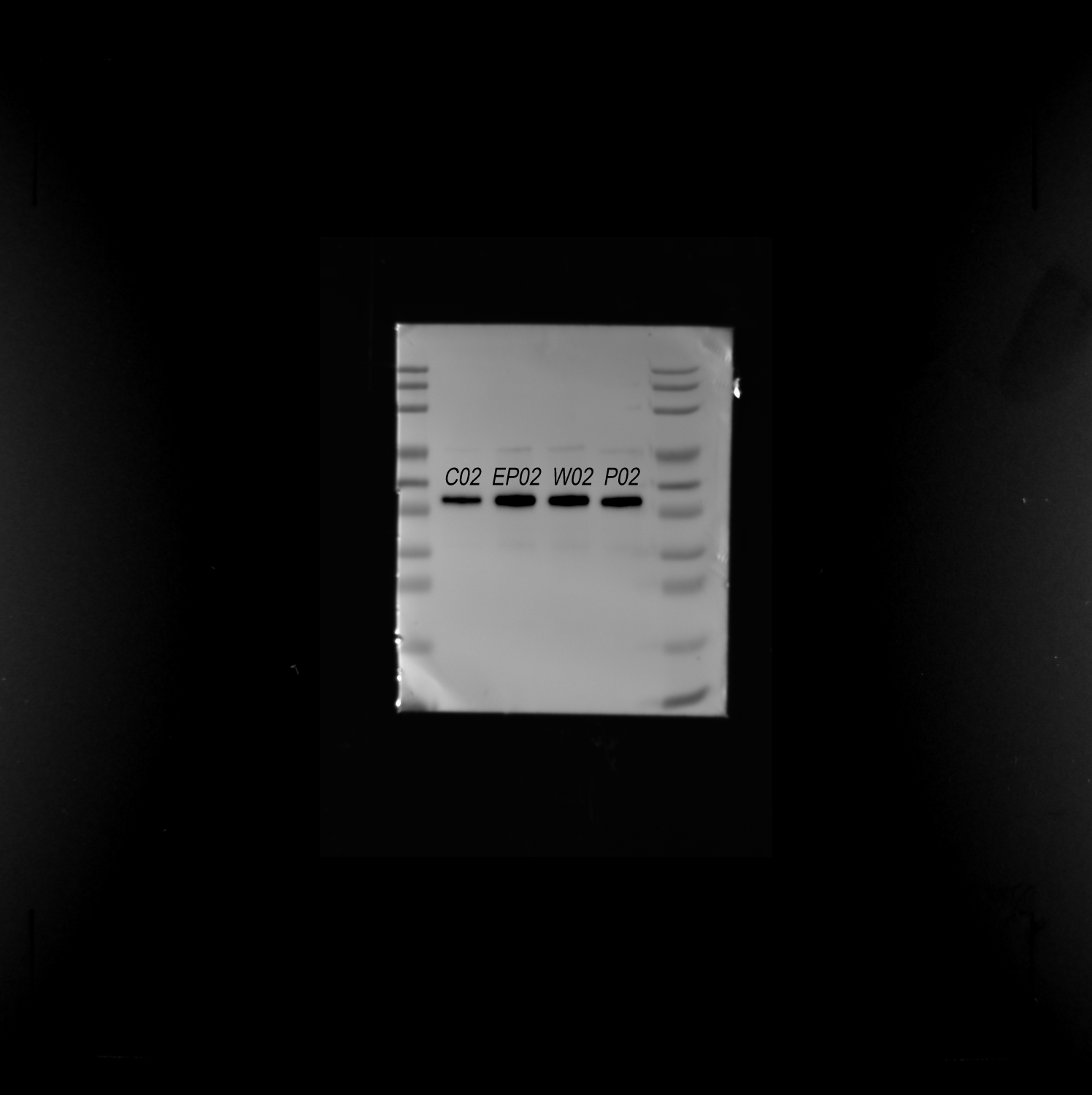

Supplement: S1 File — Contains the complete set of original, uncropped, and unprocessed Western blot images from which the data presented in this study were derived. The lanes containing the molecular weight markers are present. The corresponding molecular weight calibration reference for these markers is provided in S2 File. (ZIP) [file pone.0340313.s001.zip › WB-raw-images/Membrane2_PMEK_annotated_C02-EP02-W02-P02_original.tif]

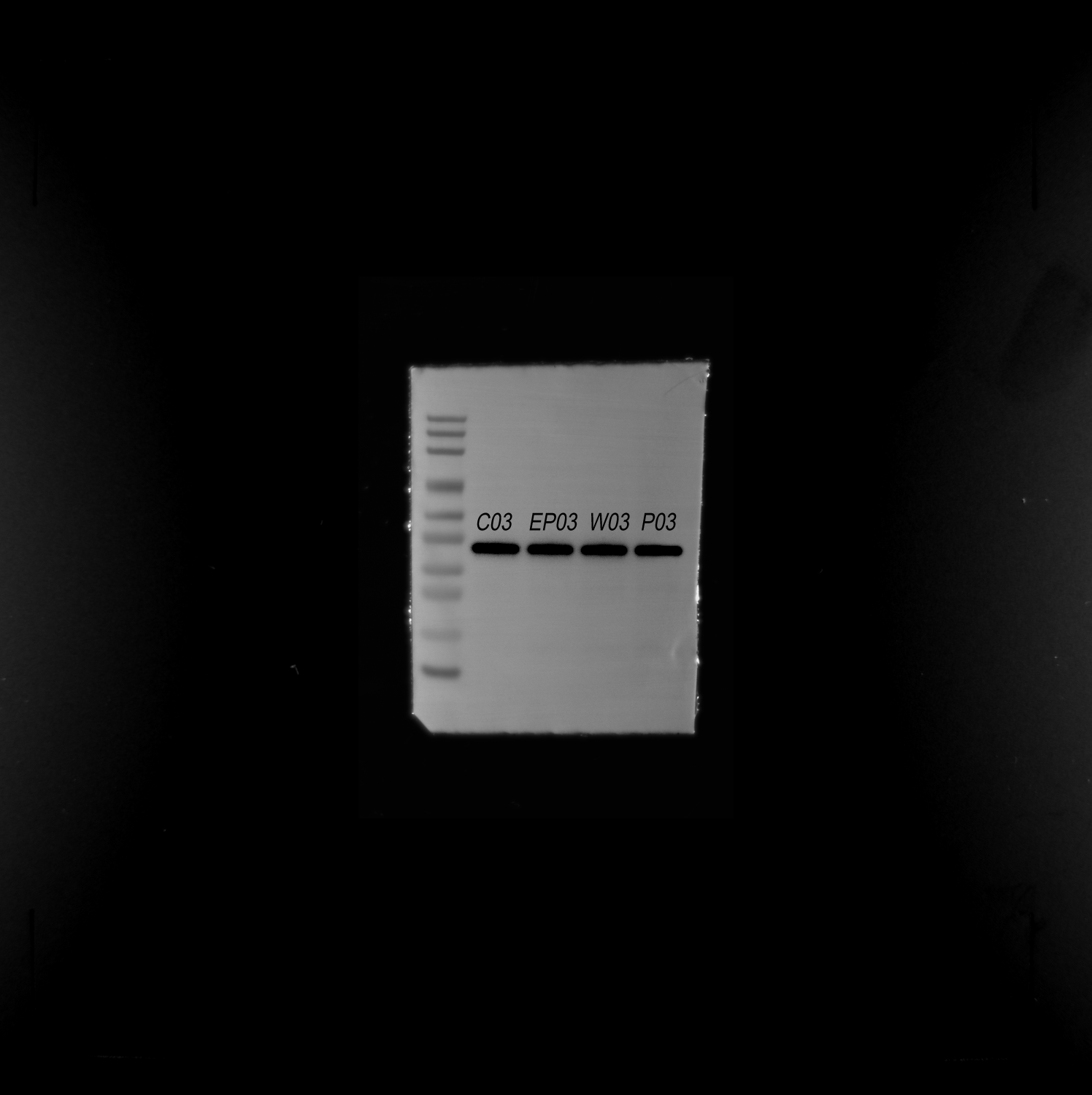

Supplement: S1 File — Contains the complete set of original, uncropped, and unprocessed Western blot images from which the data presented in this study were derived. The lanes containing the molecular weight markers are present. The corresponding molecular weight calibration reference for these markers is provided in S2 File. (ZIP) [file pone.0340313.s001.zip › WB-raw-images/Membrane3_GAPDH_annotated_C03-EP03-W03-P03_original.tif]

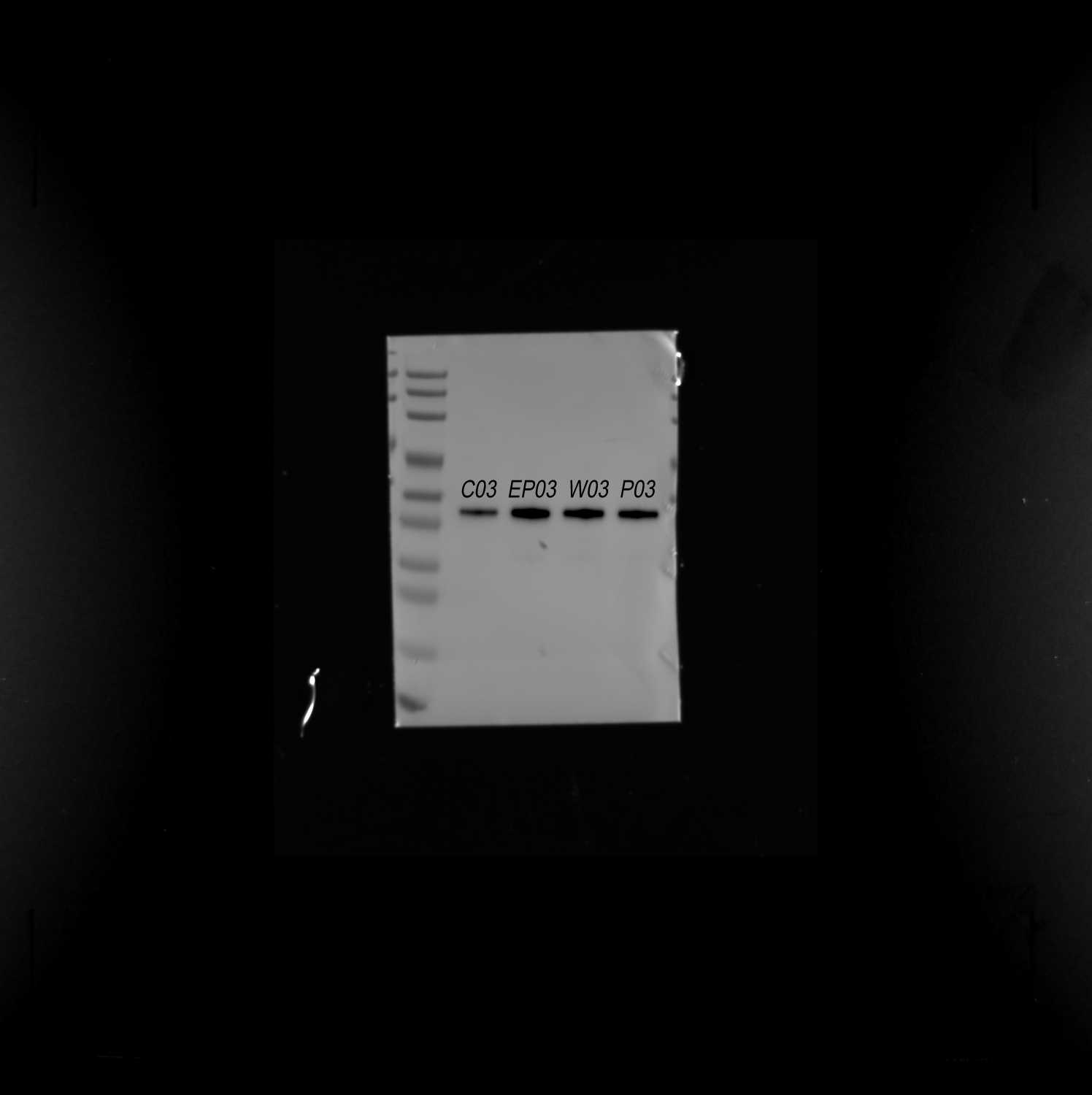

Supplement: S1 File — Contains the complete set of original, uncropped, and unprocessed Western blot images from which the data presented in this study were derived. The lanes containing the molecular weight markers are present. The corresponding molecular weight calibration reference for these markers is provided in S2 File. (ZIP) [file pone.0340313.s001.zip › WB-raw-images/Membrane3_PMEK_annotated_C03-EP03-W03-P03_original.tif]

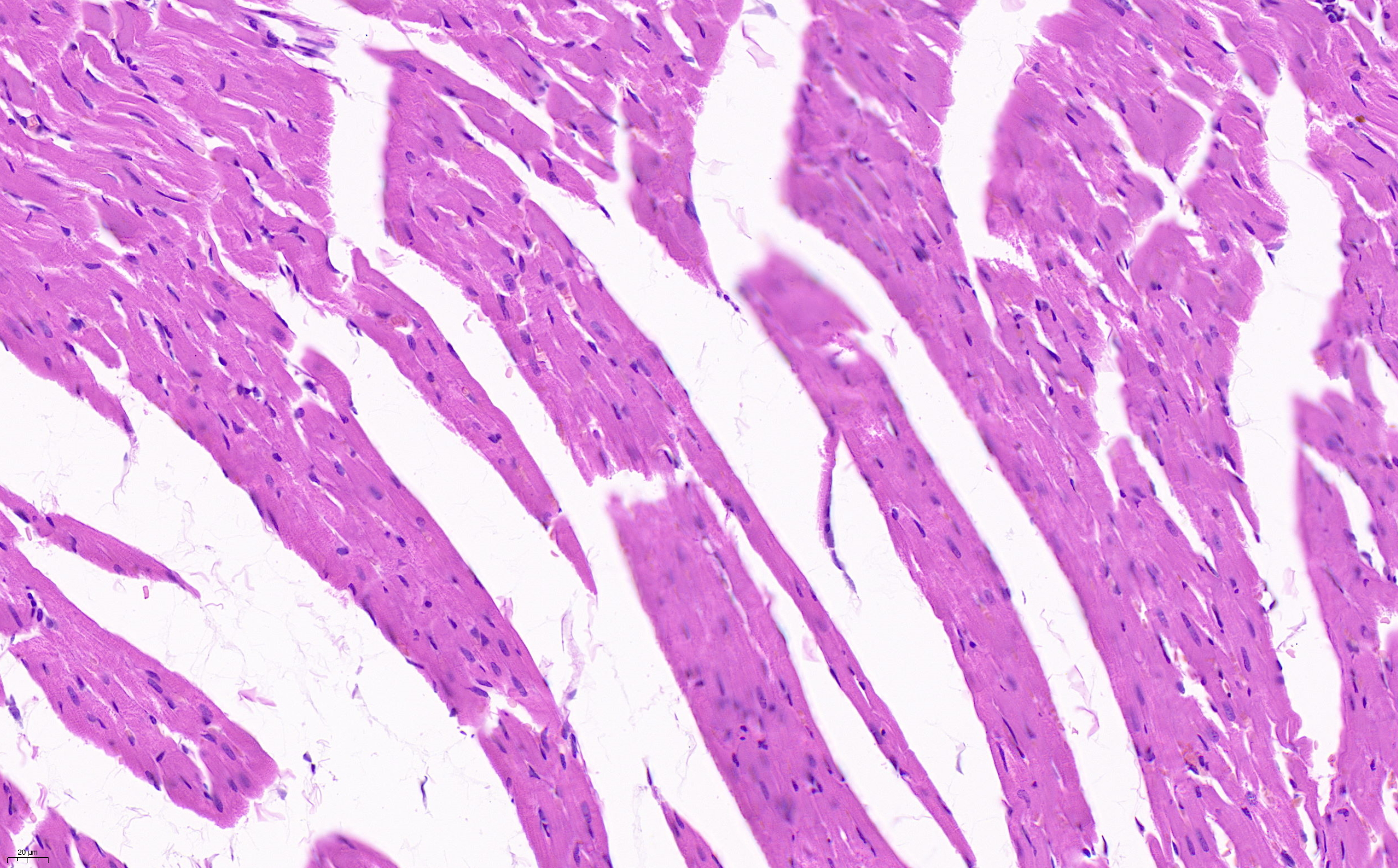

Supplement: S3 File — Contains all original, uncropped microscope images supporting the histology results in Fig 1B, Fig 2C, and Fig 3D, provided as a ZIP archive (S3_File RAW HE.zip). The archive includes the following files, with their specific correspondences detailed below: S3 File C.jpg: The 40x field for the Control group panel in Fig 1B. S3 File EP 1.jpg: The 40x field for the EP group panel in Fig 1B. S3 File EP 2.jpg: The 40x field for the EP group panel in Fig 2C. S3 File EP 3.jpg: The 40x field for the EP group panel in Fig 3D. S3 File W.jpg: The 40x field for the EP + W146 group panel in Fig 2C. S3 File P.jpg: The 40x field for the EP + PD98059 group panel in Fig 3D. S3 File EP Source.jpg: The low-magnification (3x) source image from which the three EP group 40x fields (EP_1, EP_2, EP_3) were cropped. S3 File EP Guide.tif: An annotated guide image. The locations of the cropped 40x fields are outlined and labeled as EP_1 (for Fig 1B), EP_2 (for Fig 2C), and EP_3 (for Fig 3D) within this source image. (ZIP) [file pone.0340313.s003.zip › S3_File_RAW_HE/S3_File_C.jpg.jpg]

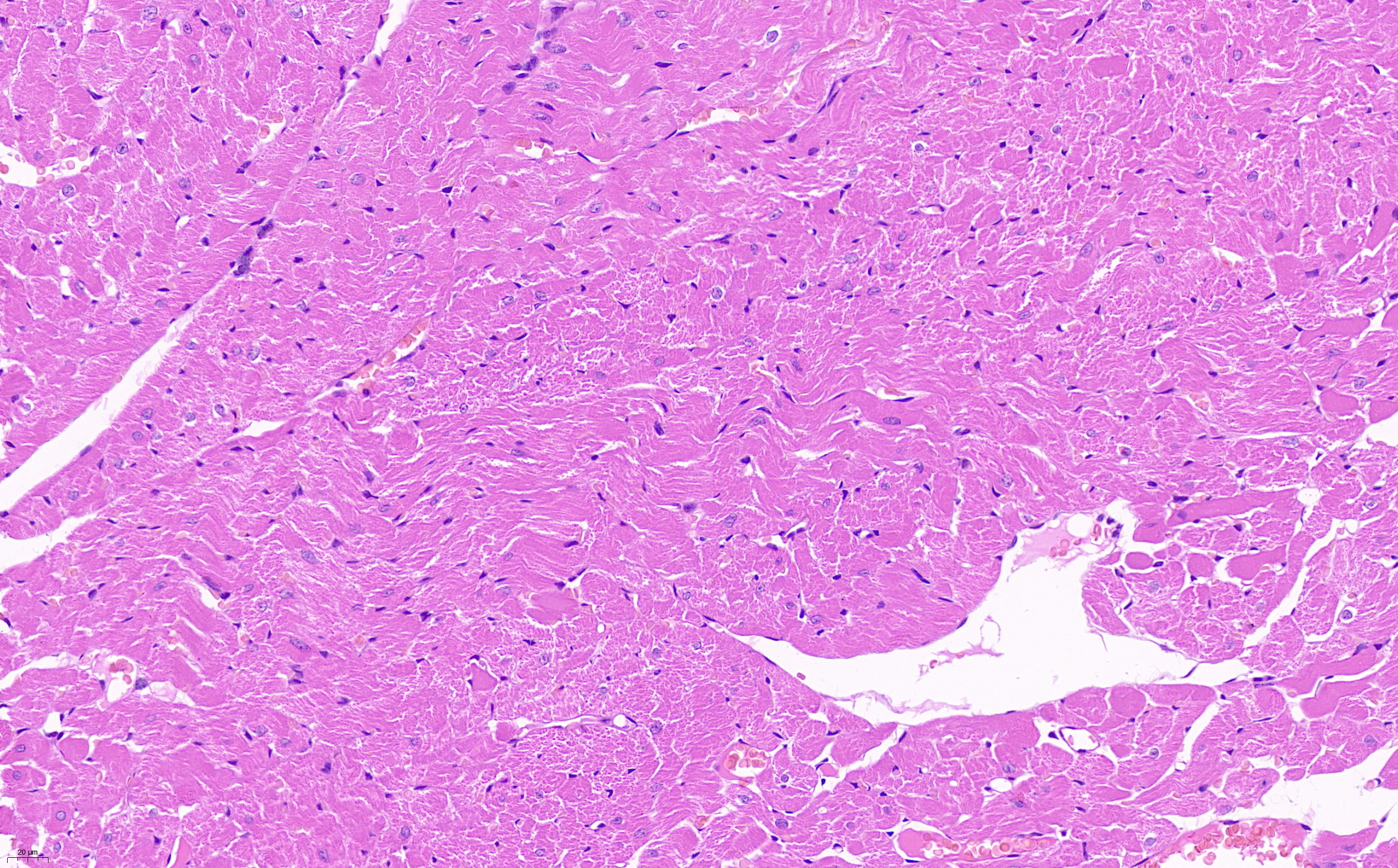

Supplement: S3 File — Contains all original, uncropped microscope images supporting the histology results in Fig 1B, Fig 2C, and Fig 3D, provided as a ZIP archive (S3_File RAW HE.zip). The archive includes the following files, with their specific correspondences detailed below: S3 File C.jpg: The 40x field for the Control group panel in Fig 1B. S3 File EP 1.jpg: The 40x field for the EP group panel in Fig 1B. S3 File EP 2.jpg: The 40x field for the EP group panel in Fig 2C. S3 File EP 3.jpg: The 40x field for the EP group panel in Fig 3D. S3 File W.jpg: The 40x field for the EP + W146 group panel in Fig 2C. S3 File P.jpg: The 40x field for the EP + PD98059 group panel in Fig 3D. S3 File EP Source.jpg: The low-magnification (3x) source image from which the three EP group 40x fields (EP_1, EP_2, EP_3) were cropped. S3 File EP Guide.tif: An annotated guide image. The locations of the cropped 40x fields are outlined and labeled as EP_1 (for Fig 1B), EP_2 (for Fig 2C), and EP_3 (for Fig 3D) within this source image. (ZIP) [file pone.0340313.s003.zip › S3_File_RAW_HE/S3_File_EP_1.jpg.jpg]

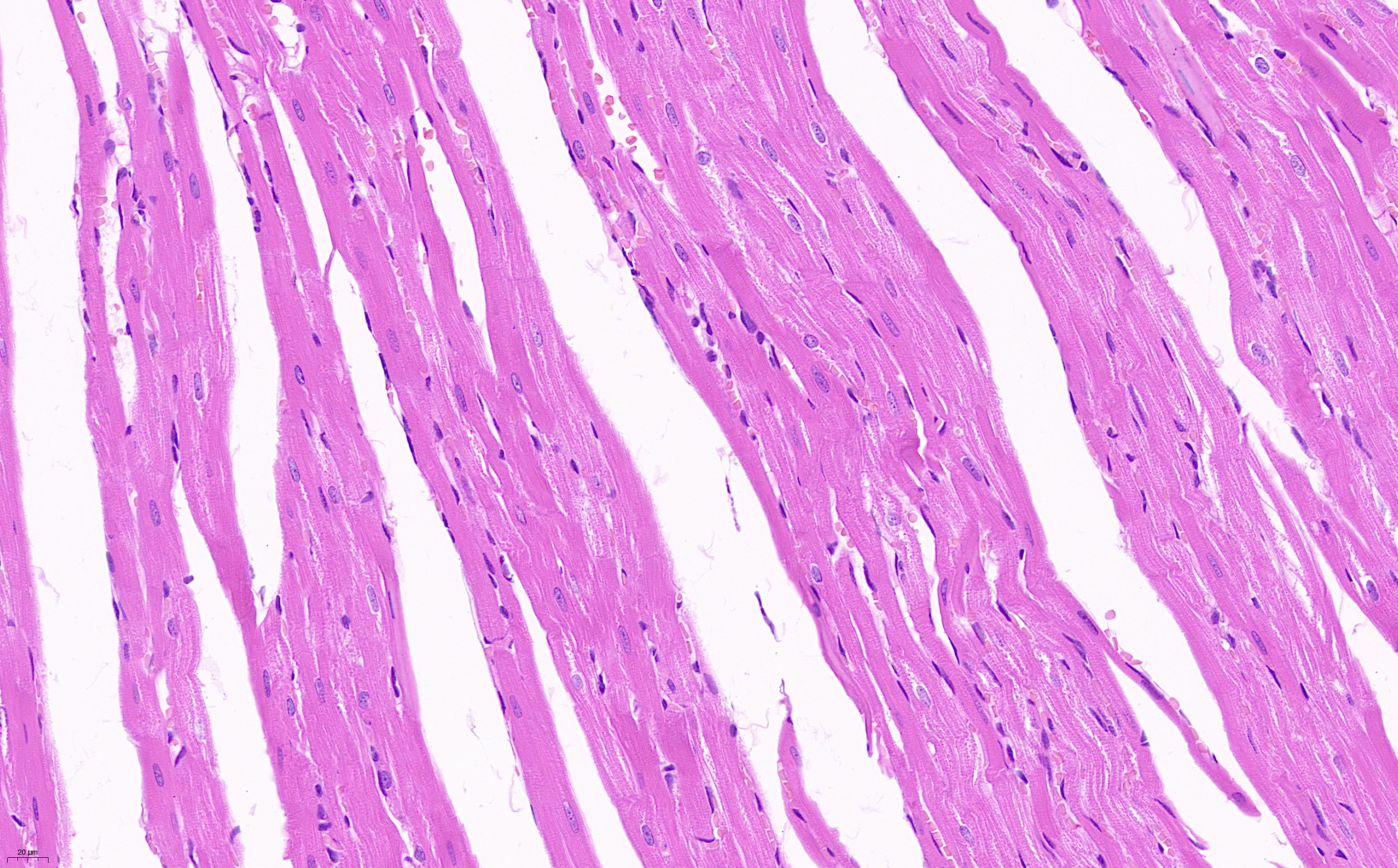

Supplement: S3 File — Contains all original, uncropped microscope images supporting the histology results in Fig 1B, Fig 2C, and Fig 3D, provided as a ZIP archive (S3_File RAW HE.zip). The archive includes the following files, with their specific correspondences detailed below: S3 File C.jpg: The 40x field for the Control group panel in Fig 1B. S3 File EP 1.jpg: The 40x field for the EP group panel in Fig 1B. S3 File EP 2.jpg: The 40x field for the EP group panel in Fig 2C. S3 File EP 3.jpg: The 40x field for the EP group panel in Fig 3D. S3 File W.jpg: The 40x field for the EP + W146 group panel in Fig 2C. S3 File P.jpg: The 40x field for the EP + PD98059 group panel in Fig 3D. S3 File EP Source.jpg: The low-magnification (3x) source image from which the three EP group 40x fields (EP_1, EP_2, EP_3) were cropped. S3 File EP Guide.tif: An annotated guide image. The locations of the cropped 40x fields are outlined and labeled as EP_1 (for Fig 1B), EP_2 (for Fig 2C), and EP_3 (for Fig 3D) within this source image. (ZIP) [file pone.0340313.s003.zip › S3_File_RAW_HE/S3_File_EP_2.jpg.jpg]

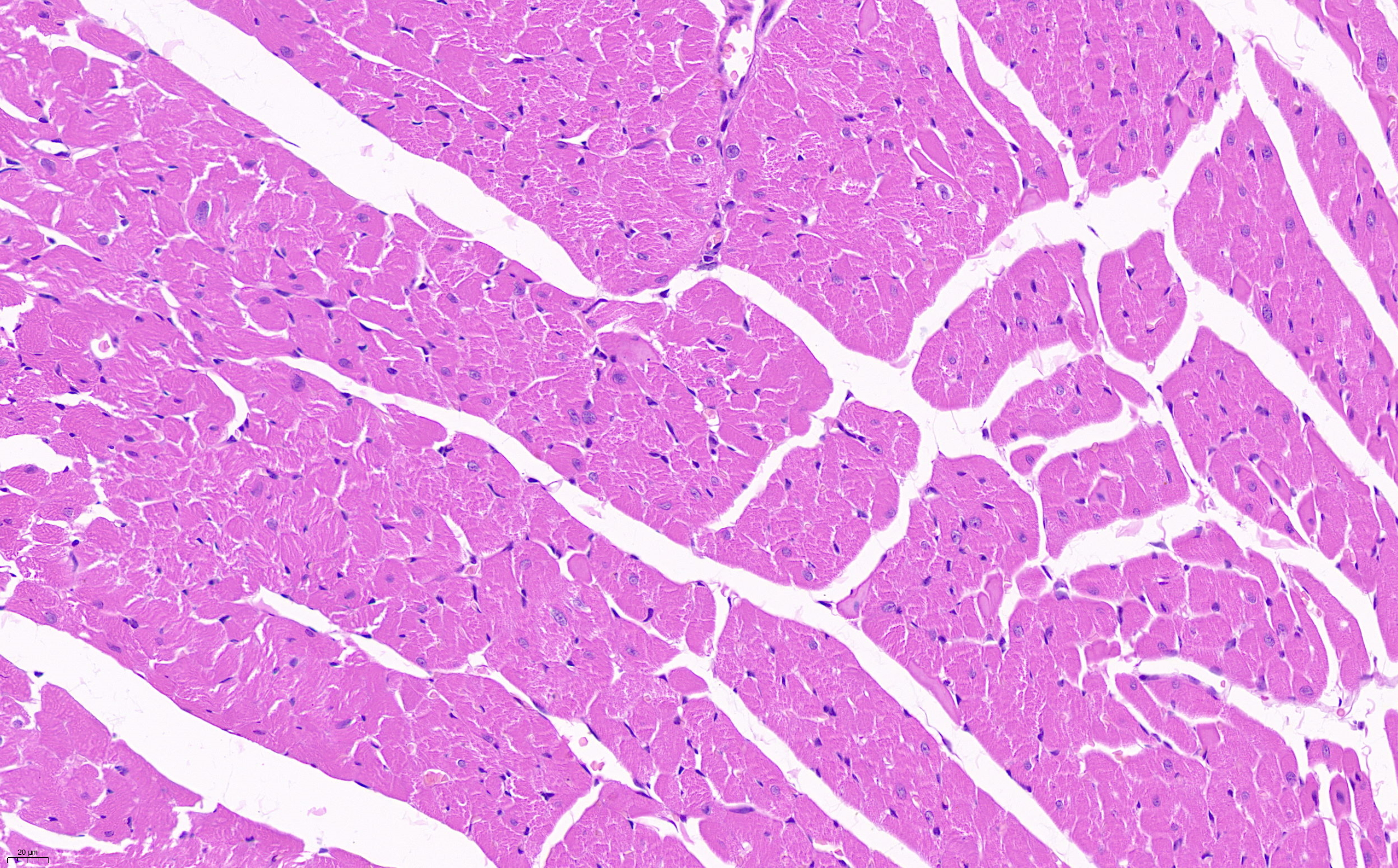

Supplement: S3 File — Contains all original, uncropped microscope images supporting the histology results in Fig 1B, Fig 2C, and Fig 3D, provided as a ZIP archive (S3_File RAW HE.zip). The archive includes the following files, with their specific correspondences detailed below: S3 File C.jpg: The 40x field for the Control group panel in Fig 1B. S3 File EP 1.jpg: The 40x field for the EP group panel in Fig 1B. S3 File EP 2.jpg: The 40x field for the EP group panel in Fig 2C. S3 File EP 3.jpg: The 40x field for the EP group panel in Fig 3D. S3 File W.jpg: The 40x field for the EP + W146 group panel in Fig 2C. S3 File P.jpg: The 40x field for the EP + PD98059 group panel in Fig 3D. S3 File EP Source.jpg: The low-magnification (3x) source image from which the three EP group 40x fields (EP_1, EP_2, EP_3) were cropped. S3 File EP Guide.tif: An annotated guide image. The locations of the cropped 40x fields are outlined and labeled as EP_1 (for Fig 1B), EP_2 (for Fig 2C), and EP_3 (for Fig 3D) within this source image. (ZIP) [file pone.0340313.s003.zip › S3_File_RAW_HE/S3_File_EP_3.jpg.jpg]

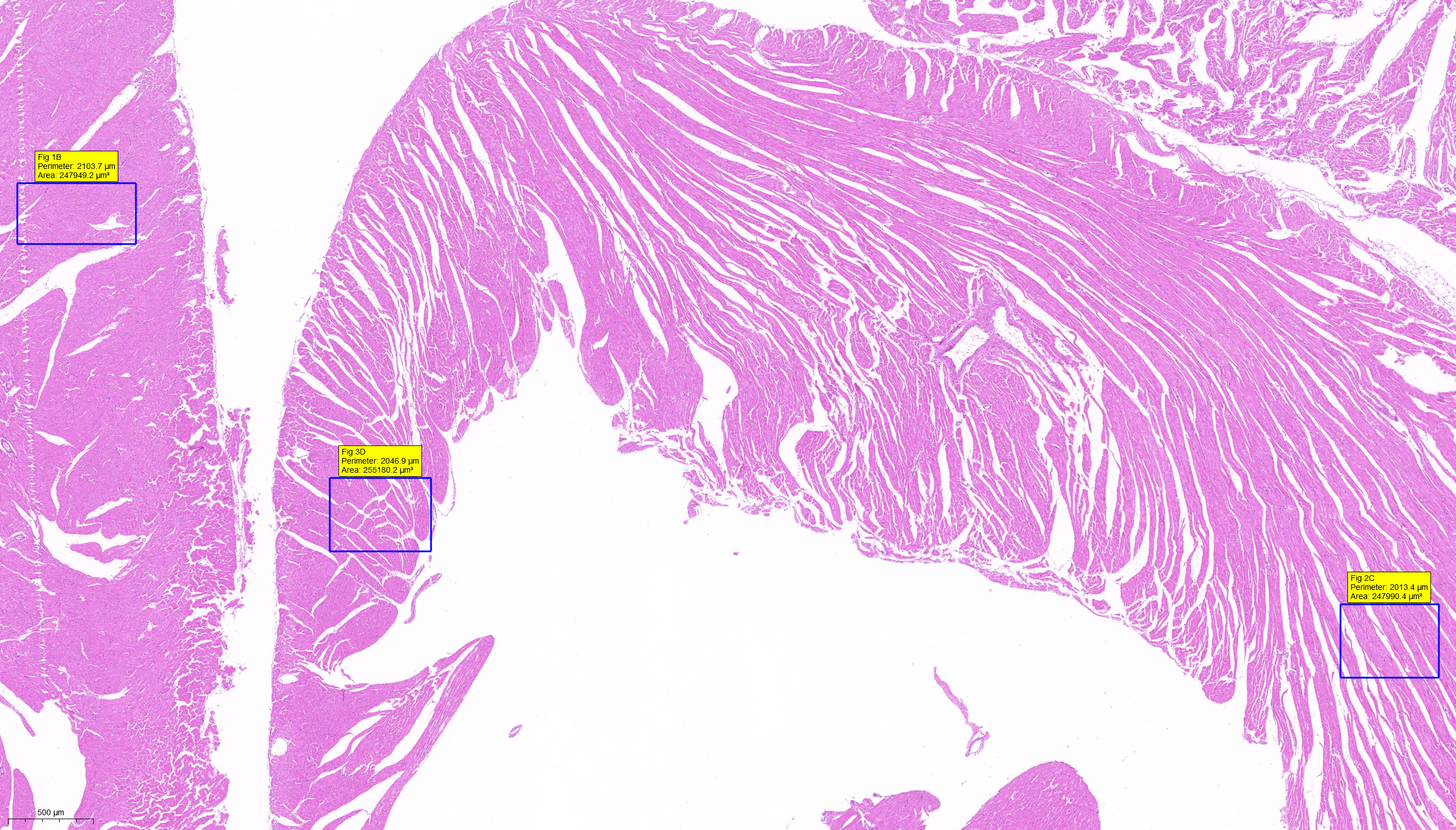

Supplement: S3 File — Contains all original, uncropped microscope images supporting the histology results in Fig 1B, Fig 2C, and Fig 3D, provided as a ZIP archive (S3_File RAW HE.zip). The archive includes the following files, with their specific correspondences detailed below: S3 File C.jpg: The 40x field for the Control group panel in Fig 1B. S3 File EP 1.jpg: The 40x field for the EP group panel in Fig 1B. S3 File EP 2.jpg: The 40x field for the EP group panel in Fig 2C. S3 File EP 3.jpg: The 40x field for the EP group panel in Fig 3D. S3 File W.jpg: The 40x field for the EP + W146 group panel in Fig 2C. S3 File P.jpg: The 40x field for the EP + PD98059 group panel in Fig 3D. S3 File EP Source.jpg: The low-magnification (3x) source image from which the three EP group 40x fields (EP_1, EP_2, EP_3) were cropped. S3 File EP Guide.tif: An annotated guide image. The locations of the cropped 40x fields are outlined and labeled as EP_1 (for Fig 1B), EP_2 (for Fig 2C), and EP_3 (for Fig 3D) within this source image. (ZIP) [file pone.0340313.s003.zip › S3_File_RAW_HE/S3_File_EP_Guide.tif.tif]

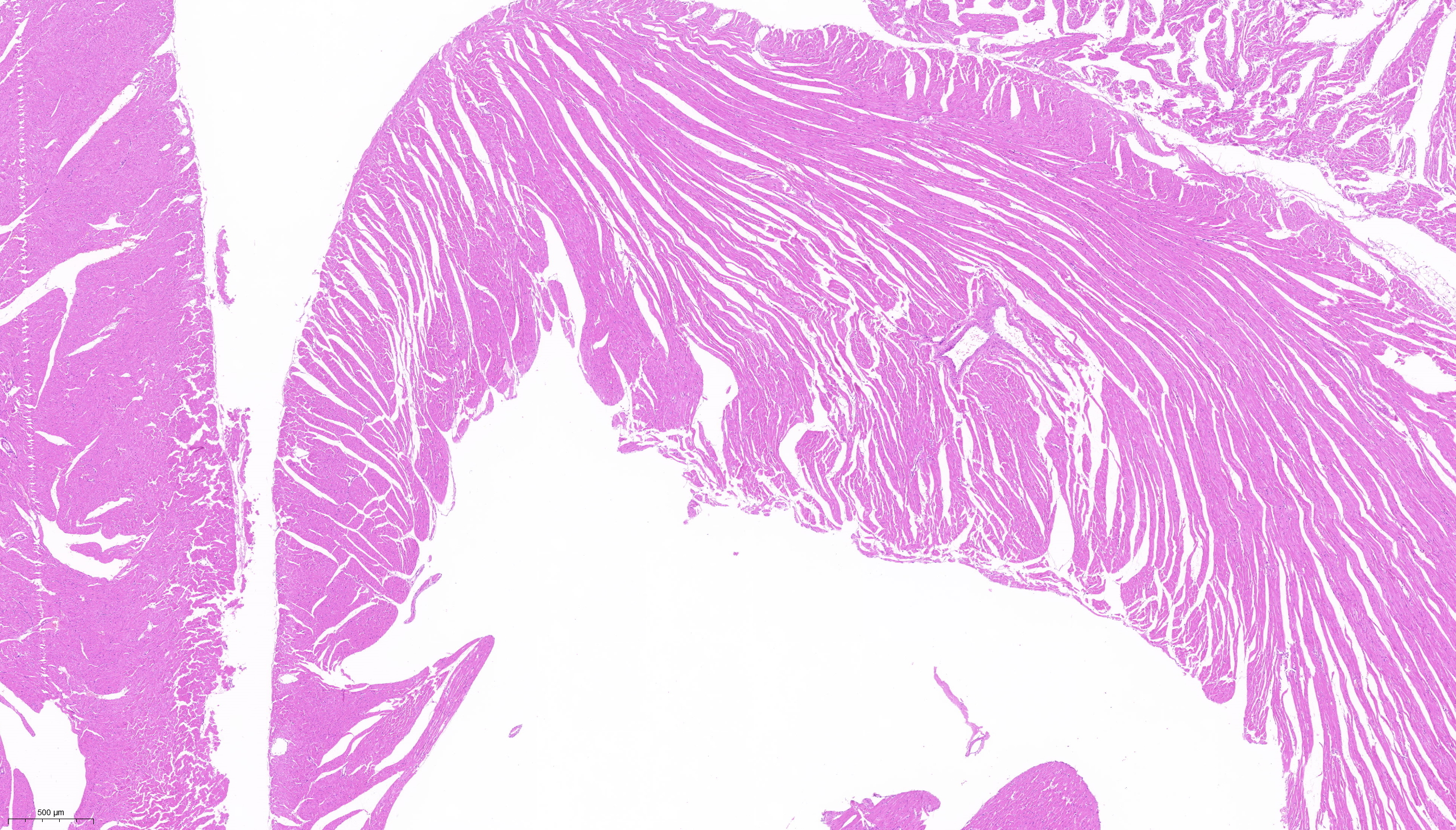

Supplement: S3 File — Contains all original, uncropped microscope images supporting the histology results in Fig 1B, Fig 2C, and Fig 3D, provided as a ZIP archive (S3_File RAW HE.zip). The archive includes the following files, with their specific correspondences detailed below: S3 File C.jpg: The 40x field for the Control group panel in Fig 1B. S3 File EP 1.jpg: The 40x field for the EP group panel in Fig 1B. S3 File EP 2.jpg: The 40x field for the EP group panel in Fig 2C. S3 File EP 3.jpg: The 40x field for the EP group panel in Fig 3D. S3 File W.jpg: The 40x field for the EP + W146 group panel in Fig 2C. S3 File P.jpg: The 40x field for the EP + PD98059 group panel in Fig 3D. S3 File EP Source.jpg: The low-magnification (3x) source image from which the three EP group 40x fields (EP_1, EP_2, EP_3) were cropped. S3 File EP Guide.tif: An annotated guide image. The locations of the cropped 40x fields are outlined and labeled as EP_1 (for Fig 1B), EP_2 (for Fig 2C), and EP_3 (for Fig 3D) within this source image. (ZIP) [file pone.0340313.s003.zip › S3_File_RAW_HE/S3_File_EP_Source.jpg.jpg]

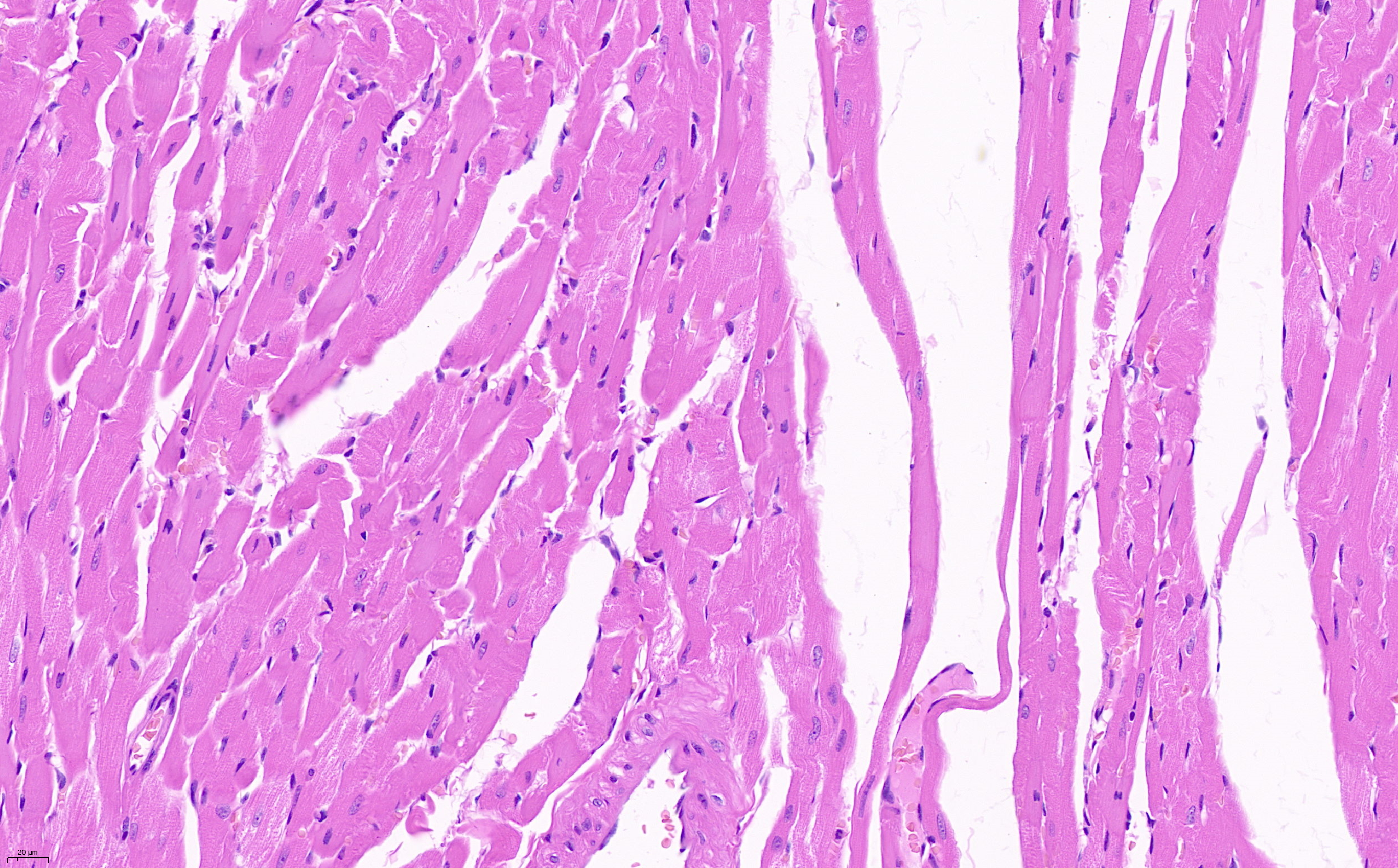

Supplement: S3 File — Contains all original, uncropped microscope images supporting the histology results in Fig 1B, Fig 2C, and Fig 3D, provided as a ZIP archive (S3_File RAW HE.zip). The archive includes the following files, with their specific correspondences detailed below: S3 File C.jpg: The 40x field for the Control group panel in Fig 1B. S3 File EP 1.jpg: The 40x field for the EP group panel in Fig 1B. S3 File EP 2.jpg: The 40x field for the EP group panel in Fig 2C. S3 File EP 3.jpg: The 40x field for the EP group panel in Fig 3D. S3 File W.jpg: The 40x field for the EP + W146 group panel in Fig 2C. S3 File P.jpg: The 40x field for the EP + PD98059 group panel in Fig 3D. S3 File EP Source.jpg: The low-magnification (3x) source image from which the three EP group 40x fields (EP_1, EP_2, EP_3) were cropped. S3 File EP Guide.tif: An annotated guide image. The locations of the cropped 40x fields are outlined and labeled as EP_1 (for Fig 1B), EP_2 (for Fig 2C), and EP_3 (for Fig 3D) within this source image. (ZIP) [file pone.0340313.s003.zip › S3_File_RAW_HE/S3_File_P.jpg.jpg]

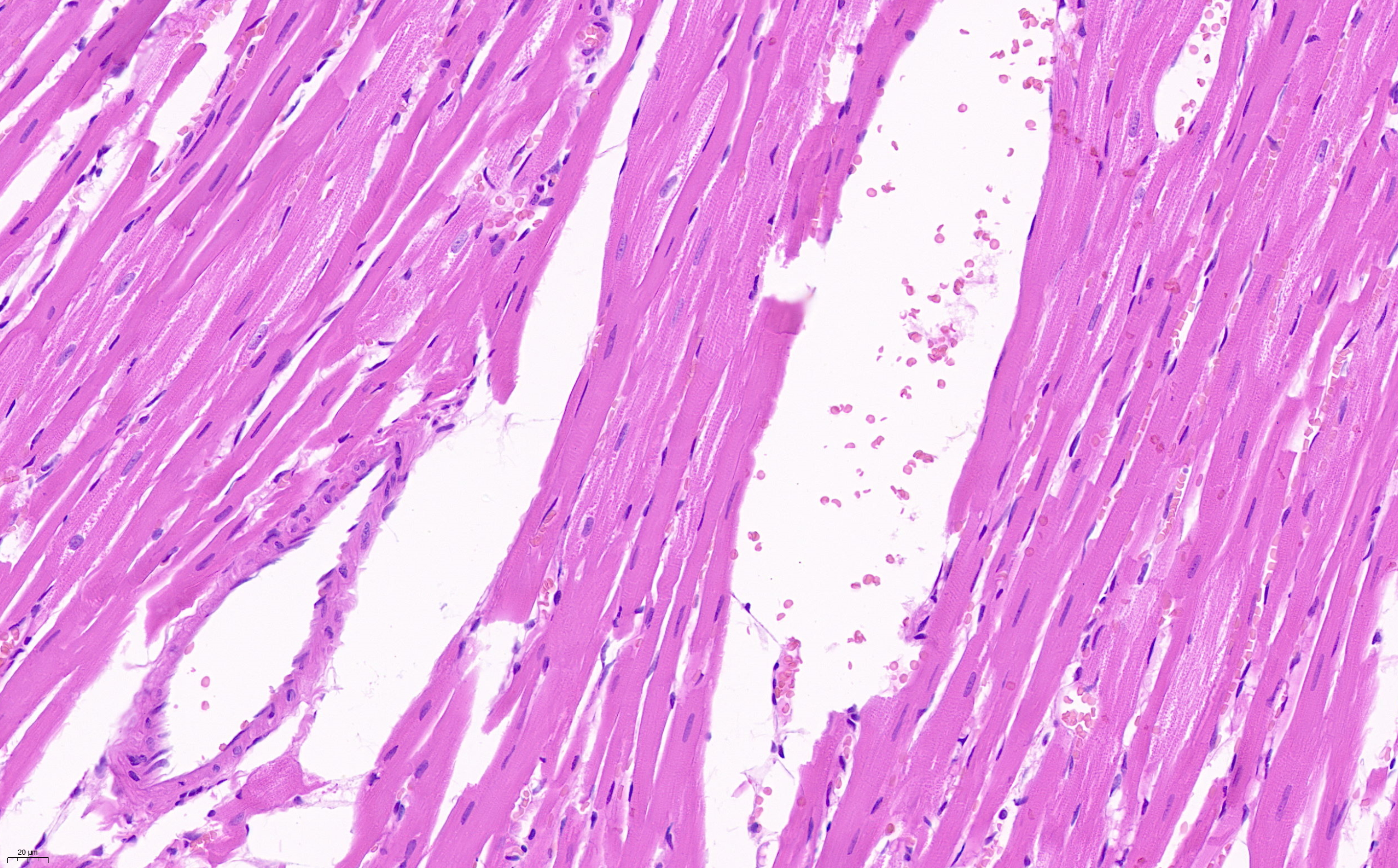

Supplement: S3 File — Contains all original, uncropped microscope images supporting the histology results in Fig 1B, Fig 2C, and Fig 3D, provided as a ZIP archive (S3_File RAW HE.zip). The archive includes the following files, with their specific correspondences detailed below: S3 File C.jpg: The 40x field for the Control group panel in Fig 1B. S3 File EP 1.jpg: The 40x field for the EP group panel in Fig 1B. S3 File EP 2.jpg: The 40x field for the EP group panel in Fig 2C. S3 File EP 3.jpg: The 40x field for the EP group panel in Fig 3D. S3 File W.jpg: The 40x field for the EP + W146 group panel in Fig 2C. S3 File P.jpg: The 40x field for the EP + PD98059 group panel in Fig 3D. S3 File EP Source.jpg: The low-magnification (3x) source image from which the three EP group 40x fields (EP_1, EP_2, EP_3) were cropped. S3 File EP Guide.tif: An annotated guide image. The locations of the cropped 40x fields are outlined and labeled as EP_1 (for Fig 1B), EP_2 (for Fig 2C), and EP_3 (for Fig 3D) within this source image. (ZIP) [file pone.0340313.s003.zip › S3_File_RAW_HE/S3_File_W.jpg.jpg]

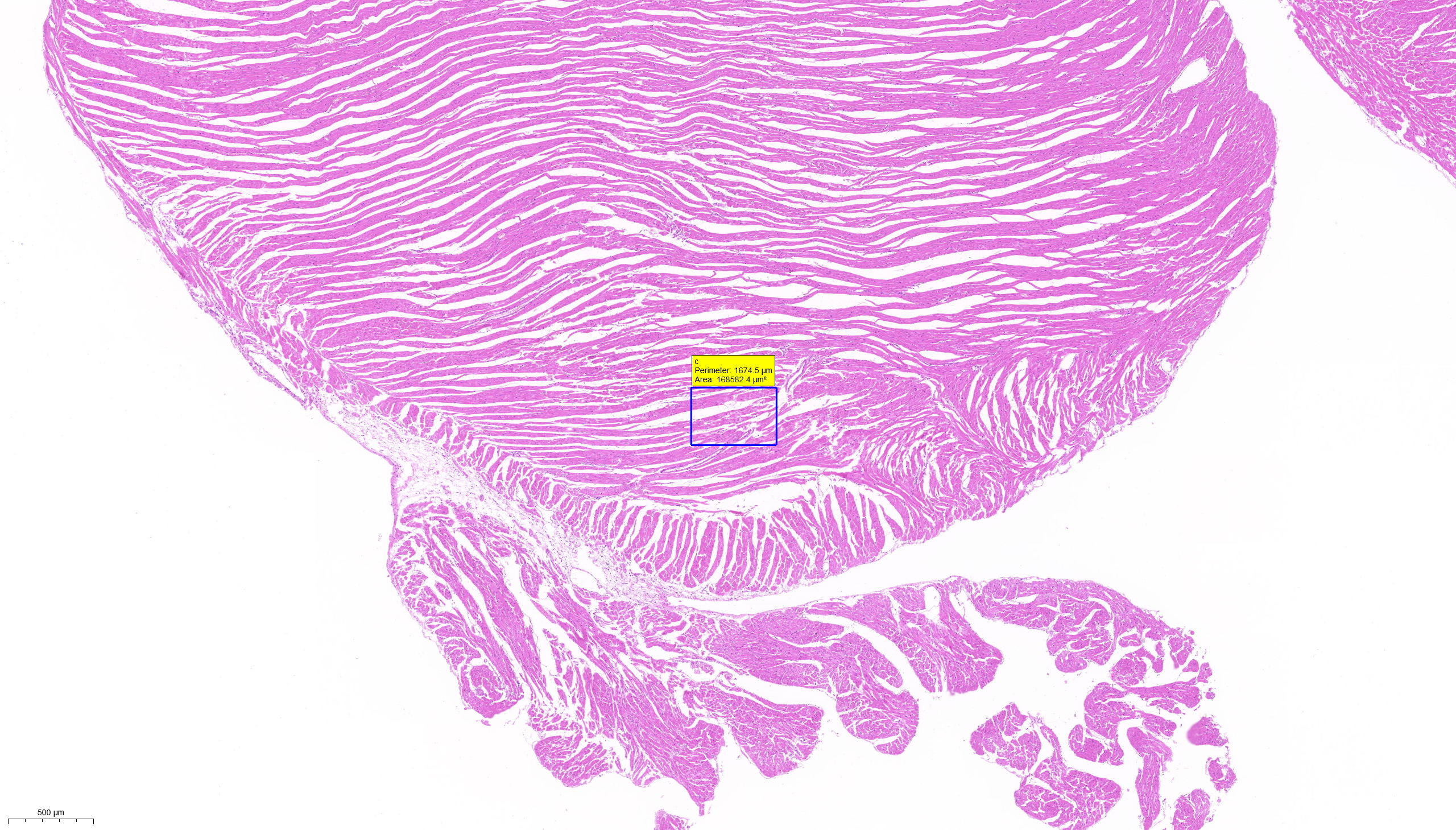

Supplement: S4 File — This file contains additional, representative H&E-stained images from the study that were not featured in the main figures but provide further context and demonstrate the consistency of observations within each experimental group. The images cover all four groups: Control (C), EP group (EP), EP + W146 (W), and EP + PD98059(P). Both low-magnification (3x) overviews and high-magnification (40x) detail views are included where available. These supplementary images support the robustness and generalizability of the histological findings presented in the manuscript. (ZIP) [file pone.0340313.s004.zip › S4_File_Supplementary_HE/S4_Suppl_C_3x_overview.jpg]

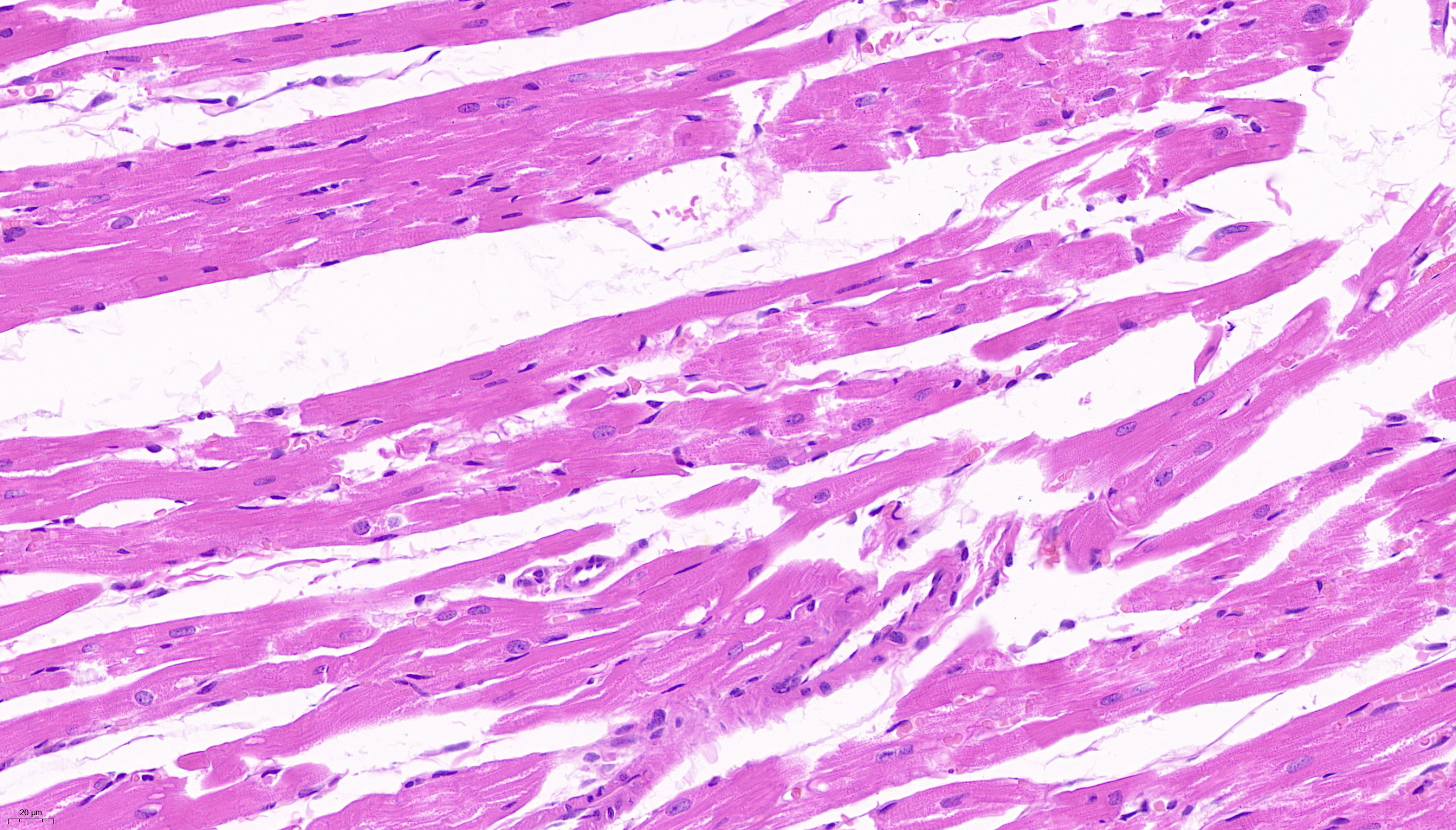

Supplement: S4 File — This file contains additional, representative H&E-stained images from the study that were not featured in the main figures but provide further context and demonstrate the consistency of observations within each experimental group. The images cover all four groups: Control (C), EP group (EP), EP + W146 (W), and EP + PD98059(P). Both low-magnification (3x) overviews and high-magnification (40x) detail views are included where available. These supplementary images support the robustness and generalizability of the histological findings presented in the manuscript. (ZIP) [file pone.0340313.s004.zip › S4_File_Supplementary_HE/S4_Suppl_C_40x_detail.jpg]

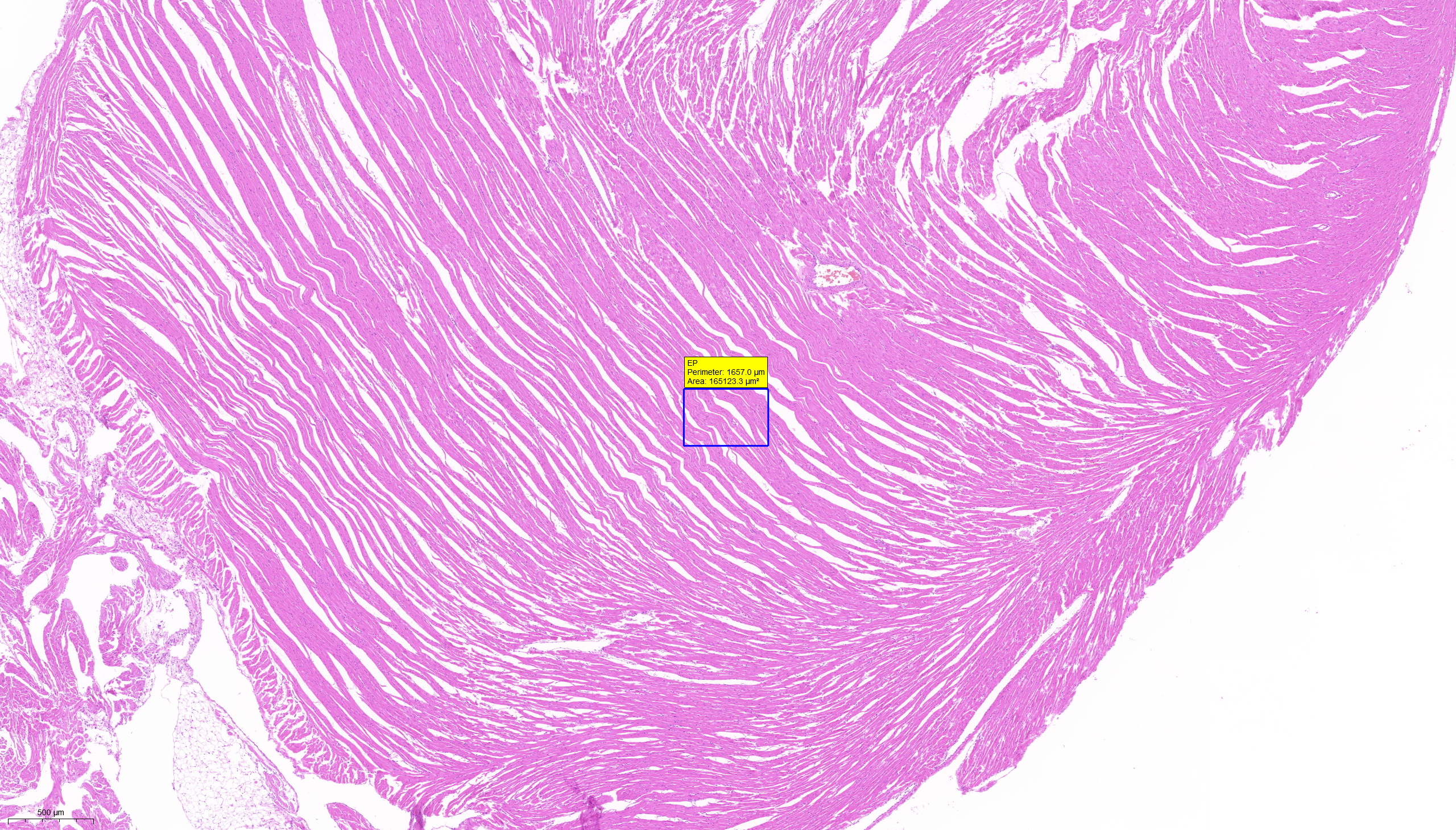

Supplement: S4 File — This file contains additional, representative H&E-stained images from the study that were not featured in the main figures but provide further context and demonstrate the consistency of observations within each experimental group. The images cover all four groups: Control (C), EP group (EP), EP + W146 (W), and EP + PD98059(P). Both low-magnification (3x) overviews and high-magnification (40x) detail views are included where available. These supplementary images support the robustness and generalizability of the histological findings presented in the manuscript. (ZIP) [file pone.0340313.s004.zip › S4_File_Supplementary_HE/S4_Suppl_EP_3x_overview.jpg]

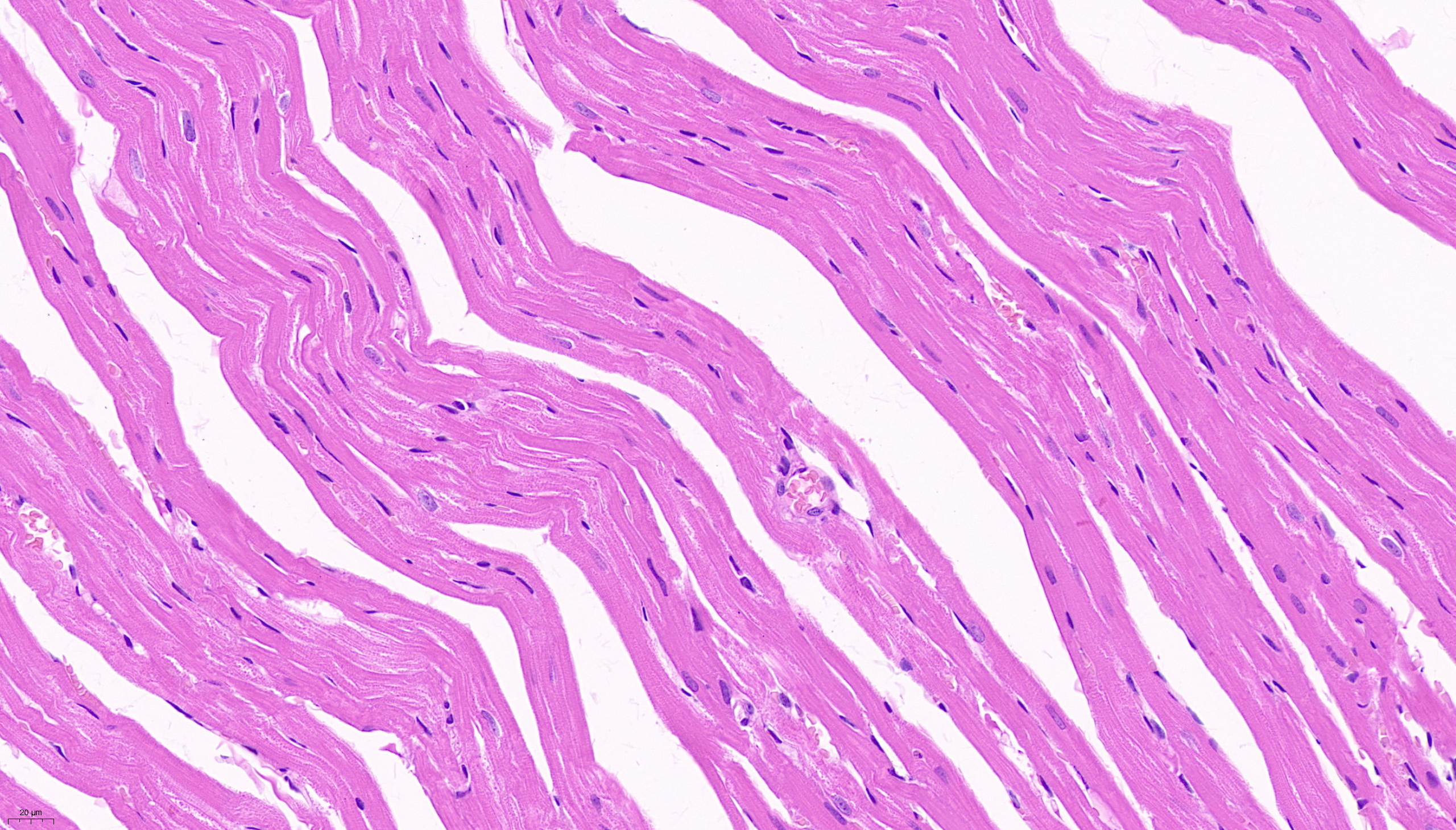

Supplement: S4 File — This file contains additional, representative H&E-stained images from the study that were not featured in the main figures but provide further context and demonstrate the consistency of observations within each experimental group. The images cover all four groups: Control (C), EP group (EP), EP + W146 (W), and EP + PD98059(P). Both low-magnification (3x) overviews and high-magnification (40x) detail views are included where available. These supplementary images support the robustness and generalizability of the histological findings presented in the manuscript. (ZIP) [file pone.0340313.s004.zip › S4_File_Supplementary_HE/S4_Suppl_EP_40x_detail.jpg]

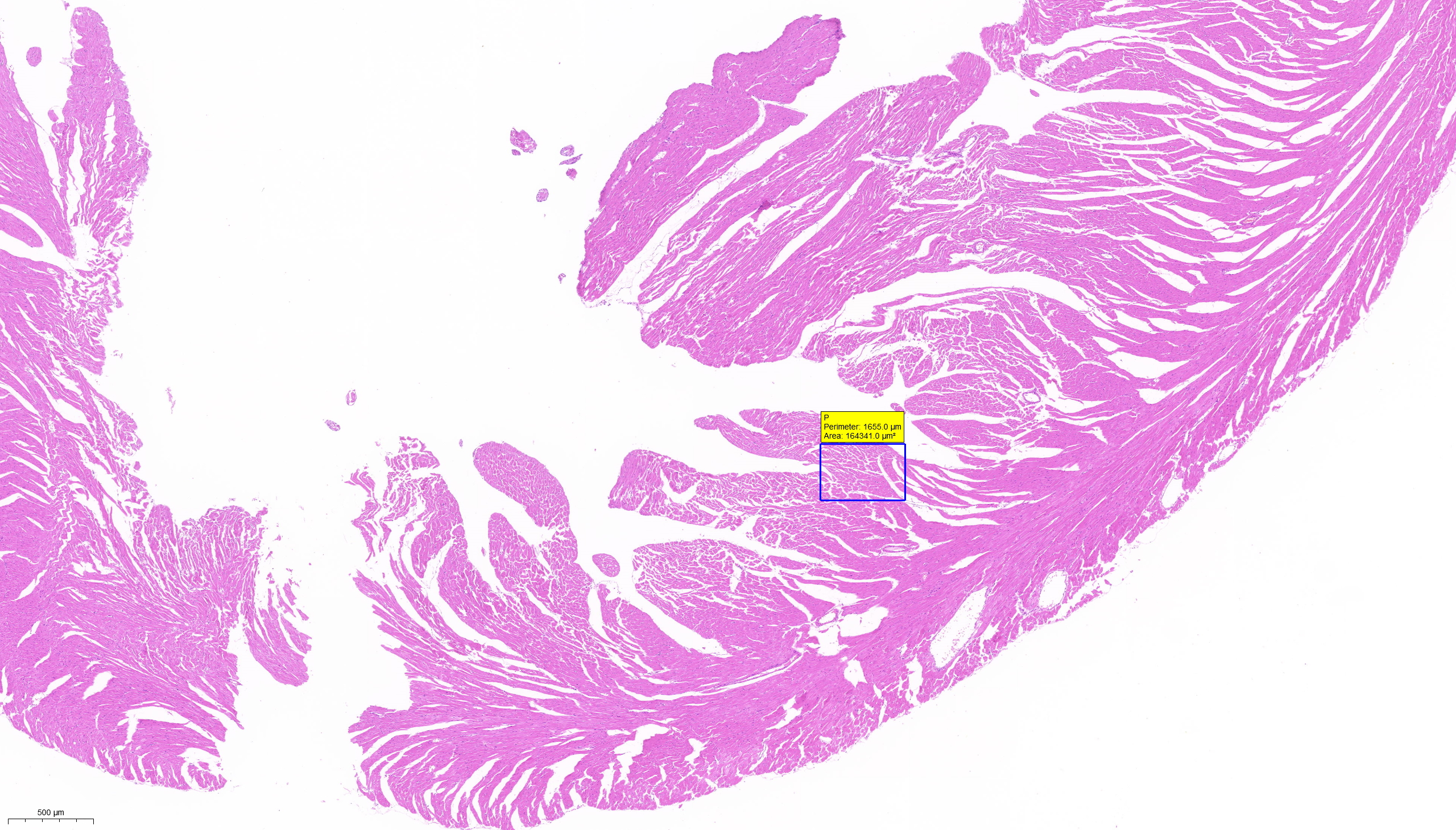

Supplement: S4 File — This file contains additional, representative H&E-stained images from the study that were not featured in the main figures but provide further context and demonstrate the consistency of observations within each experimental group. The images cover all four groups: Control (C), EP group (EP), EP + W146 (W), and EP + PD98059(P). Both low-magnification (3x) overviews and high-magnification (40x) detail views are included where available. These supplementary images support the robustness and generalizability of the histological findings presented in the manuscript. (ZIP) [file pone.0340313.s004.zip › S4_File_Supplementary_HE/S4_Suppl_P_3x_overview.jpg]

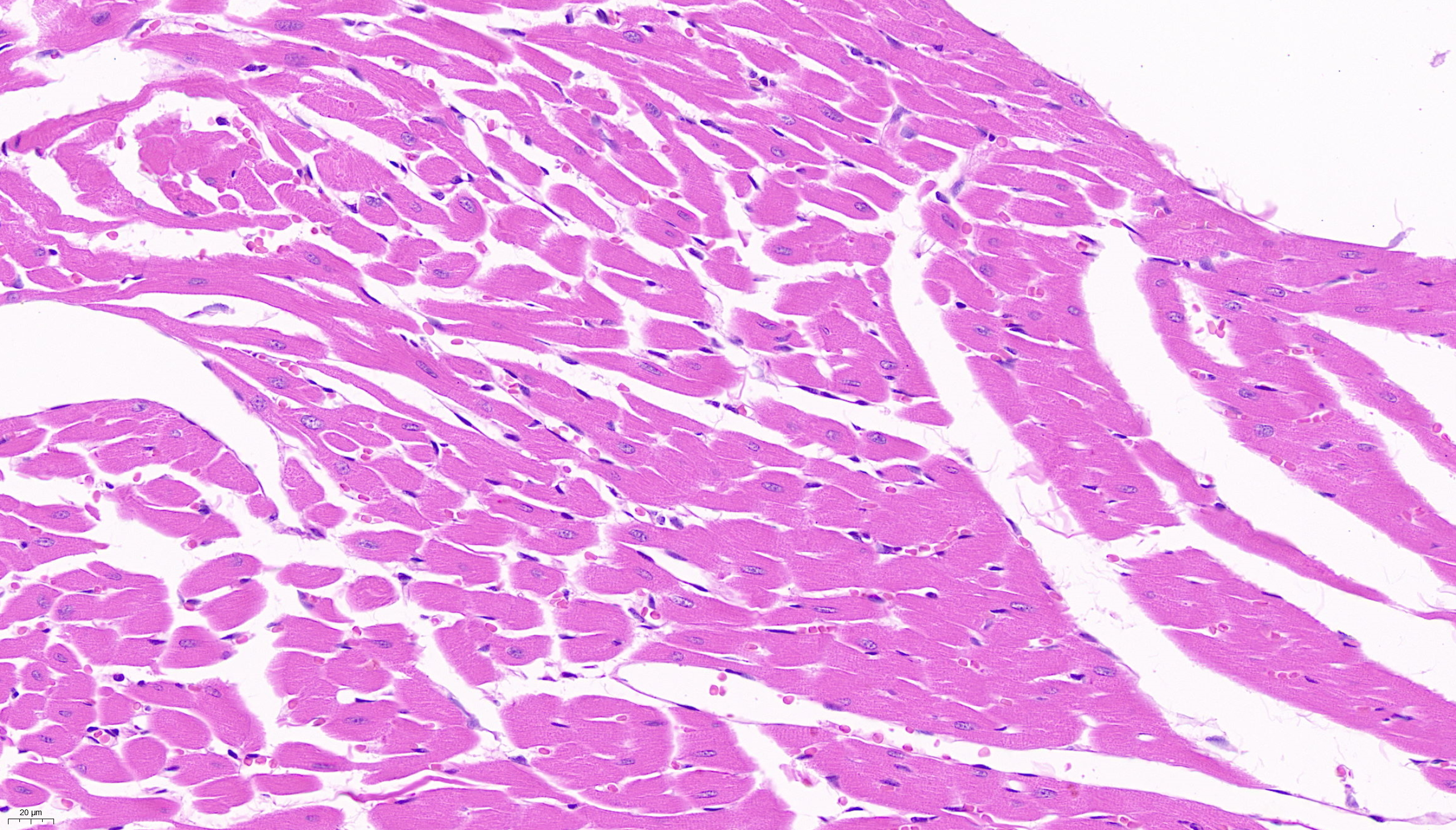

Supplement: S4 File — This file contains additional, representative H&E-stained images from the study that were not featured in the main figures but provide further context and demonstrate the consistency of observations within each experimental group. The images cover all four groups: Control (C), EP group (EP), EP + W146 (W), and EP + PD98059(P). Both low-magnification (3x) overviews and high-magnification (40x) detail views are included where available. These supplementary images support the robustness and generalizability of the histological findings presented in the manuscript. (ZIP) [file pone.0340313.s004.zip › S4_File_Supplementary_HE/S4_Suppl_P_40x_detail.jpg]

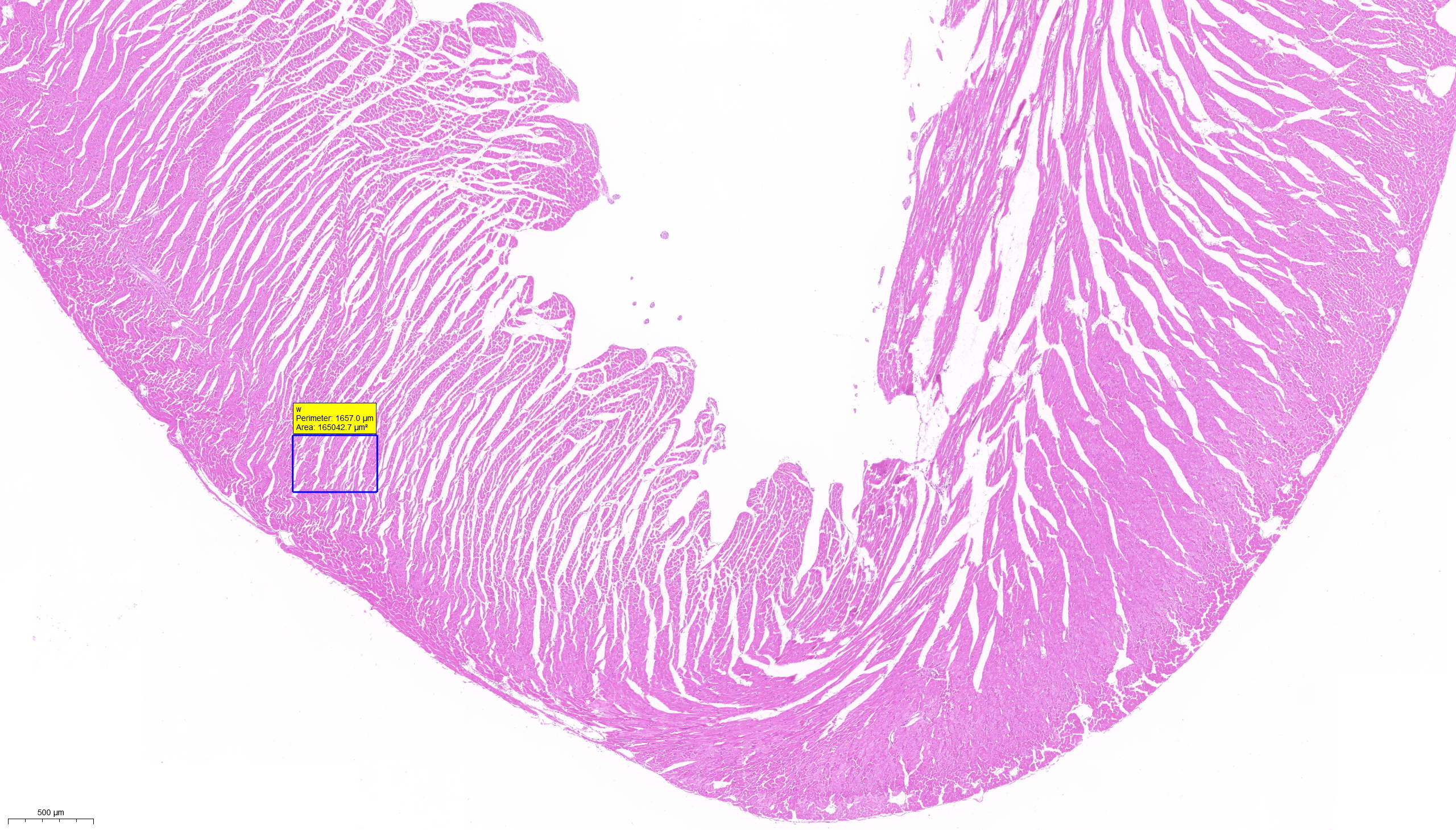

Supplement: S4 File — This file contains additional, representative H&E-stained images from the study that were not featured in the main figures but provide further context and demonstrate the consistency of observations within each experimental group. The images cover all four groups: Control (C), EP group (EP), EP + W146 (W), and EP + PD98059(P). Both low-magnification (3x) overviews and high-magnification (40x) detail views are included where available. These supplementary images support the robustness and generalizability of the histological findings presented in the manuscript. (ZIP) [file pone.0340313.s004.zip › S4_File_Supplementary_HE/S4_Suppl_W_3x_overview.jpg]

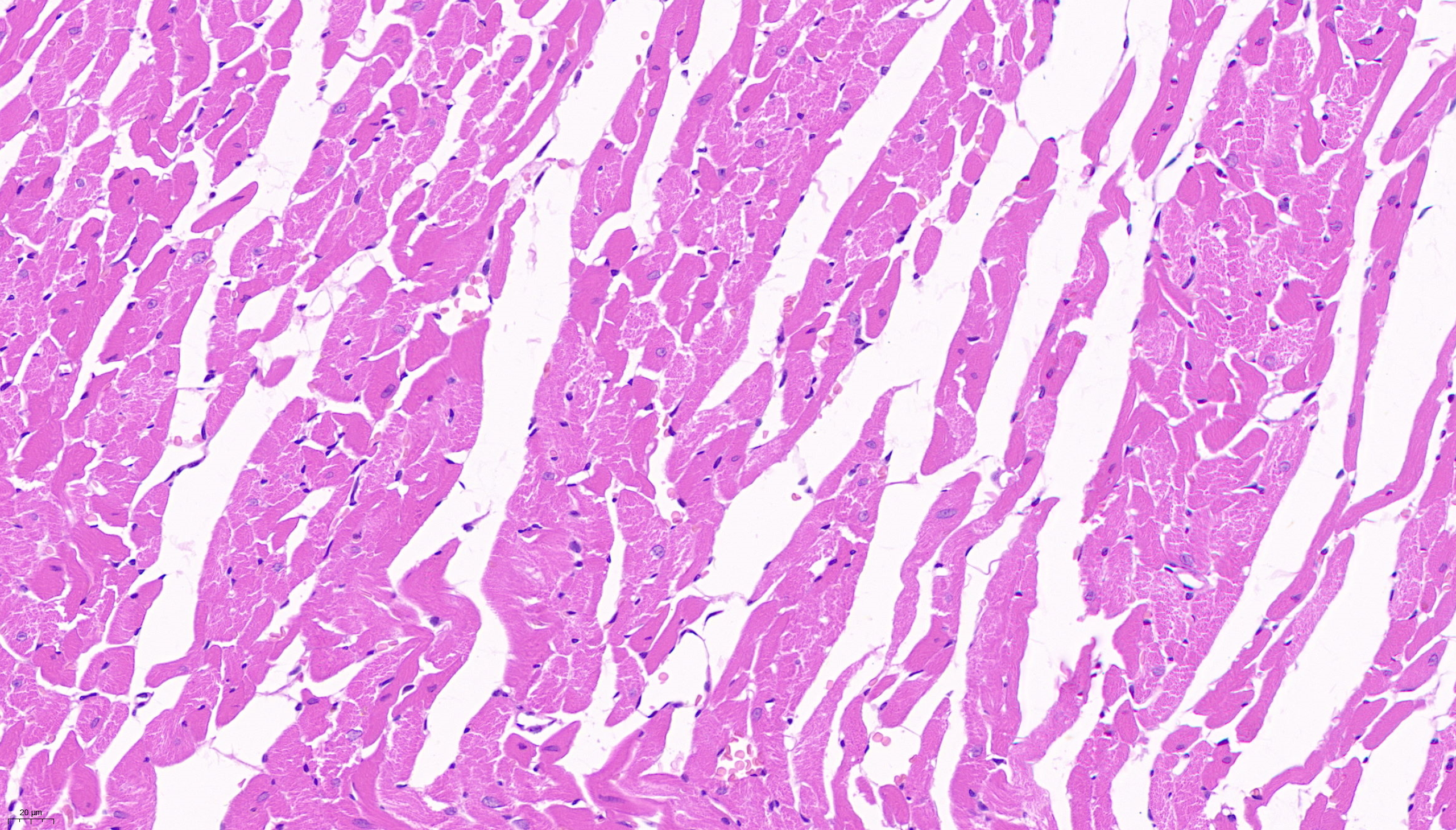

Supplement: S4 File — This file contains additional, representative H&E-stained images from the study that were not featured in the main figures but provide further context and demonstrate the consistency of observations within each experimental group. The images cover all four groups: Control (C), EP group (EP), EP + W146 (W), and EP + PD98059(P). Both low-magnification (3x) overviews and high-magnification (40x) detail views are included where available. These supplementary images support the robustness and generalizability of the histological findings presented in the manuscript. (ZIP) [file pone.0340313.s004.zip › S4_File_Supplementary_HE/S4_Suppl_W_40x_detail.jpg]
