# Supplementary material for: Effect of exercise preconditioning on myocardial content of Sphingosine1-phosphate and its mechanism in rats after exhaustive exercise
Source: PLoS One. 2026 Jan 7;21(1):e0340313. doi: 10.1371/journal.pone.0340313 (PMC12779141; doi:10.1371/journal.pone.0340313)
Supplement: S2 File — Provides a reference image showing the precise alignment of the molecular weight markers (in kDa) with their corresponding bands in the original Western blot images contained in S1 File. This allows for independent verification of protein sizes presented in the main figures. (PDF) [file pone.0340313.s002.pdf]

Western Blot

1.Antibody Information

	Primary Antibody					Secondary Antibody			
Antibody	Producer	Product Number	Molecular Weight（kDa）	Source	Dilution Ratio	Secondary Antibody	Producer	Product Number	Dilution Ratio
GAPDH	Servicebio	GB15002	37	Mouse	1: 2000	HRP-Goat anti-Mouse	Servicebio	GB23301	1：3000
P-MEK	Servicebio	GB115603-100	45	Rabbit	1:500	HRP-Goat anti-Rabbit	Servicebio	GB23303	1：5000

2.WB result and analysis：Use the AIWBwellTM analysis software to analyze the saved raw image in tiff format.

3.Analysis method introduction:
AIWBwellTM is a Western blot image analysis software based on artificial intelligence learning introduced by Servicebio. AI deep learning principle is adopted to train algorithms based on massive data and integrate them into automated image analysis software. The specific process is as follows：

(1) Automatic direction correction: automatic positioning correction image direction, can be manually adjusted according to requirements；

(2) Set molecular weight: manually mark the appropriate molecular weight；

(3) Calculation: According to the demand, the software automatically locates the strip according to the selected rectangle and calculates the gray value；

(4) Analysis: Automatically calculate the analysis results according to the original basic data and the algorithm formula, and generate reports.

4. Items of evaluation:

(1) Integrated density val: original gray value automatically read by the software, original data；

(2) ratio: The ratio of indicator grayscale value to internal parameter grayscale value, which is the relative content of the sample.

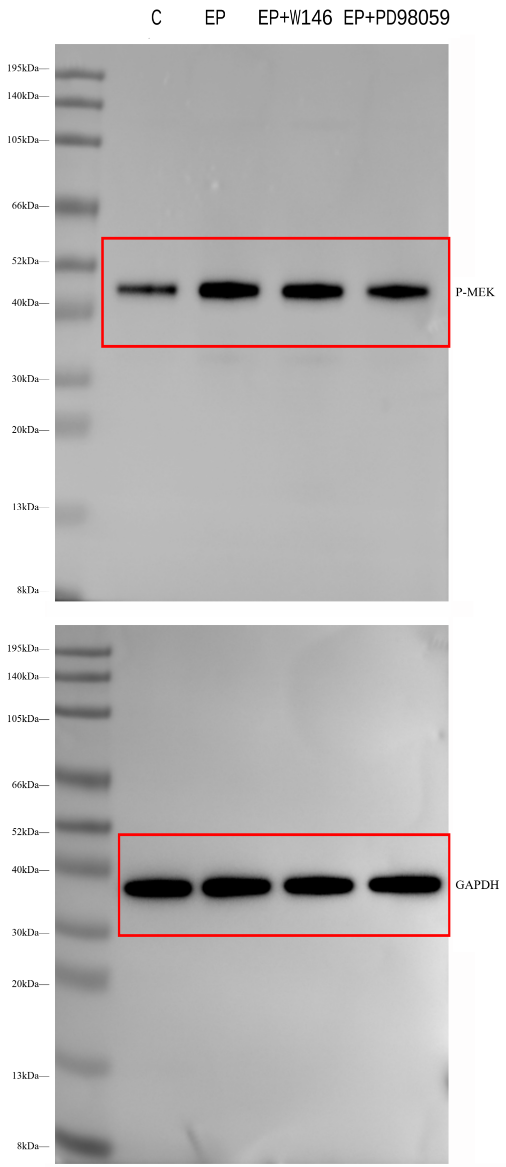
C
EP
EP+W146
EP+PD98059
195kDa
140kDa
105kDa
66kDa
52kDa
40kDa
30kDa
20kDa
13kDa
8kDa
P-MEK
195kDa
140kDa
105kDa
66kDa
52kDa
40kDa
30kDa
20kDa
13kDa
8kDa
GAPDH

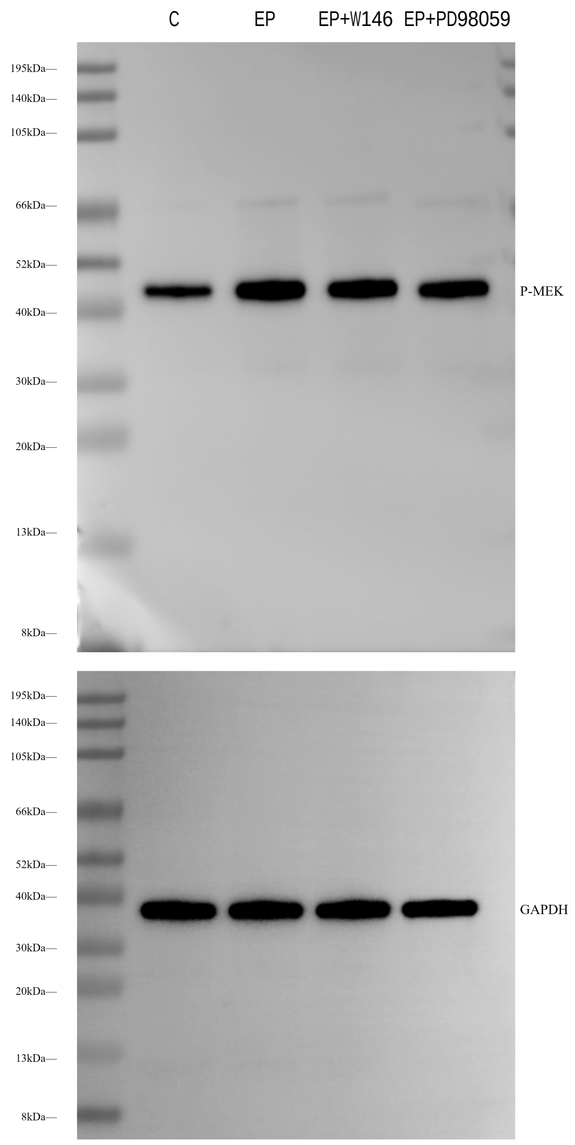
C
EP
EP+W146
EP+PD98059
195kDa
140kDa
105kDa
66kDa
52kDa
40kDa
30kDa
20kDa
13kDa
8kDa
P-MEK
195kDa
140kDa
105kDa
66kDa
52kDa
40kDa
30kDa
20kDa
13kDa
8kDa
GAPDH

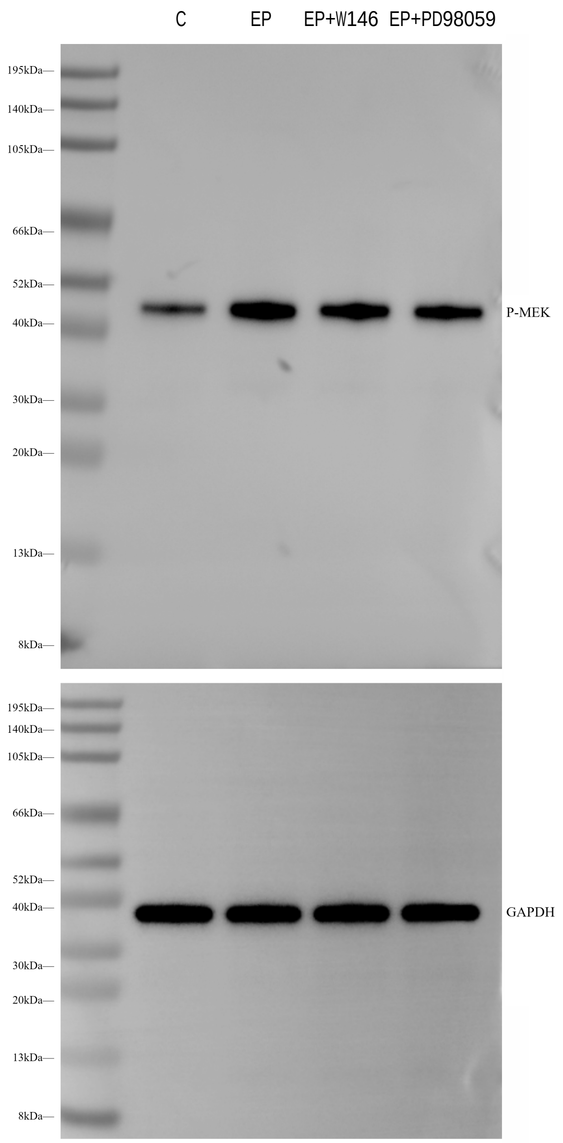

C
EP
EP+W146
EP+PD98059
195kDa
140kDa
105kDa
66kDa
52kDa
40kDa
30kDa
20kDa
13kDa
8kDa
P-MEK
195kDa
140kDa
105kDa
66kDa
52kDa
40kDa
30kDa
20kDa
13kDa
8kDa
GAPDH
