# Supplementary material for: Effect of exercise preconditioning on myocardial content of Sphingosine1-phosphate and its mechanism in rats after exhaustive exercise
Source: PLoS One. 2026 Jan 7;21(1):e0340313. doi: 10.1371/journal.pone.0340313 (PMC12779141; doi:10.1371/journal.pone.0340313)
Supplement: Attachment — Submitted filename: Response to Reviewers.docx [file pone.0340313.s006.docx]

**Dear Editors and Reviewers:**

Thank you for your letter and for the reviewers’ comments concerning our manuscript entitled “Effect of Exercise Preconditioning on Myocardial Content of S1P and its Mechanism in Rats after Exhaustive Exercise” (ID: PONE-D-25-14601R1). Those comments are all valuable and very helpful for revising and improving our paper, as well as the important guiding significance to our researches. We have studied comments carefully and have made correction which we hope meet with approval.

**SUGGESTIONS FROM EDITOR**

*1.Please ensure that your manuscript meets* *PLOS ONE's style requirements, including those for file naming. The PLOS ONE style templates can be found at*

*https://journals.plos.org/plosone/s/file?id=wjVg/PLOSOne_formatting_sample_main_body.pdf and*

*https://journals.plos.org/plosone/s/file?id=ba62/PLOSOne_formatting_sample_title_authors_affiliations.pdf*

2. *To comply with PLOS ONE submissions requirements, in your Methods section, please provide additional information regarding the experiments involving animals and ensure you have included d**etails on (1) methods of sacrifice, and (2) efforts to alleviate suffering.*

*Please note that funding information should not appear in any section or other areas of your manuscript. We will only publish funding information present in the Funding Statement section of the online submission form. Please remove any funding-related text from the manuscript.*

*4. Thank you for stating the following financial disclosure:*

*“This work was supported by the General Program of Fujian Provincial Natural Science Foundation* *(2020J011350).”*

*Please state what role the funders took in the study. If the funders had no role, please state: "The funders had no role in study design, data collection and analysis, decision to publish, or preparation of the manuscript."*

*If this statement is not correct you must amend it as needed.*

*Please include this amended Role of Funder statement in your cover letter; we will change the online submission form on your behalf.*

*5. We note that your Data Availability Statement is currently as follows: All relevant data are within the manuscript and in Supporting Information files.*

*Please confirm at this time whether or not your submission contains all raw data required to replicate the results of your study. Authors must share the “minimal data set” for their submission. PLOS defines the minimal data set to consist of the data required to replicate all study findings reported in the article, as well as related metadata and methods (https://journals.plos.org/plosone/s/data-availability#loc-minimal-data-set-definition).*

*For example, authors should submit the following data:*

*- The values behind the means, standard deviations and other measures reported;*

*- The values used to build graphs;*

*- The points extracted from images for analysis.*

*Authors do not need to submit their entire data set if only a portion of the data was used in the reported study.*

*If your submission does not contain these data, please either upload them as Supporting Information files or deposit them to a stable, public repository and provide us with the relevant URLs, DOIs, or accession numbers. For a list of recommended repositories, please see https://journals.plos.org/plosone/s/recommended-repositories.*

*If there are ethical or legal restrictions on sharing a de-identified data set, please explain them in detail (e.g., data contain potentially sensitive information, data are owned by a third-party organization, etc.) and who has imposed them (e.g., an ethics committee). Please also provide contact information for a data access committee, ethics committee, or other institutional body to which data requests may be sent. If data are owned by a third party, please indicate how others may request data access.*

*6. When completing the data availability statement of the submission form, you indicated that you will make your data available on acceptance. We strongly recommend all authors decide on a data sharing plan before acceptance, as the process can be lengthy and hold up publication timelines. Please note that, though access restrictions are acceptable now, your entire data will need to be made freely accessible if your manuscript is accepted for publication. This policy applies to all data except where public deposition would breach compliance with the protocol approved by your research ethics board. If you are unable to adhere to our open data policy, please kindly revise your statement to explain your reasoning and we will seek the editor's input on an exemption. Please be assured that, once you have provided your new statement, the assessment of your exemption will not hold up the peer review process.*

*7. Your ethics statement should only appear in the Methods section of your manuscript. If your ethics statement is written in any section besides the Methods, please move it to the Methods section and delete it from any other section. Please ensure that your ethics statement is included in your manuscript, as the ethics statement entered into the online submission form will not be published alongside your manuscript.*

*8. Please include your tables as part of your main manuscript and remove the individual files. Please note that supplementary tables (should remain/ be uploaded) as separate "supporting information" files.*

*9. PLOS ONE now requires that authors provide the original uncropped and unadjusted images underlying all blot or gel results reported in a submission’s figures or Supporting Information files. This policy and the journal’s other requirements for blot/gel reporting and figure preparation are described in detail at https://journals.plos.org/plosone/s/figures#loc-blot-and-gel-reporting-requirements and https://journals.plos.org/plosone/s/figures#loc-preparing-figures-from-image-files. When you submit your revised manuscript, please ensure that your figures adhere fully to these guidelines and provide the original underlying images for all blot or gel data reported in your submission. See the following link for instructions on providing the original image data: https://journals.plos.org/plosone/s/figures#loc-original-images-for-blots-and-gels.*

*In your cover letter, please note whether your blot/gel image data are in Supporting Information or posted at a public data repository, provide the repository URL if relevant, and provide specific details as to which raw blot/gel images, if any, are not available. Email us at plosone@plos.org if you have any questions.*

10. *If the reviewer comments include a recommendation to cite specific previously published works, please review and evaluate these publications to determine whether they are relevant and should be cited. There is no requirement to cite these works unless the editor has indicated otherwise.*

Yes, we have check our manuscript meets the*PLOS ONE's style requirements.*We are sorry for write the wrong funders numeber (2020J011350), the correct number is 2024J01941(P17). Revised portion are marked in red in the paper. The main corrections in the paper and the responds to the reviewer’s comments are as flowing:

**Reviewer #1**: The authors are presenting a rodent study in which the want to describe the effect of Exercise Preconditioning on Myocardial Content of S1P and its Mechanism after Exhaustive Exercise. Unfortunately, the data presented is not comprehensive enough to seriously underline the authors' conclusions. There is a clear need for improvement both in the methodology and in the presentation and interpretation of the data. Furthermore, the knowledge gap to be filled here is very small. There is some data on S1P in exercise and training that should have been discussed in more detail here. Overall, the idea is a good one, but significant improvements are needed before publication. My suggestions are as follows:

***Abstract:***

***- Typos in S1P***

***- Methods described in great detail in relation to the rest.***

***- The results are difficult to understand. The enumeration (1.,2.,3.) seems more like a bullet point list. Where possible, the wording should also be chosen so that results are compared with the control group. Otherwise this is very misleading.***

We have re-written this part according to the Reviewer’s suggestion.

***Methods:***

***- The headings should be more clearly separated from the text. Reading would be easier if the headings were also visually marked (bold, italics, etc.).***

***- W146 is not just an “S1P receptor blocker”, but a specific antagonist of S1PR1. This should be made clear, as S1P can mediate its effects via the other S1PRs***

*We have made correction according to the Reviewer’s comments.*

*-* ***The description of PD98059 as a MAPK blocker is also not ideal. It is a MAPKK inhibitor (MEK1/2)***

***- It remains unclear in which solvent the substances were administered***

All the reagents are prepared using normal saline as the solvent.

*-* ***Were there any differences in the duration of the final “single bout of exhaustive swimming exercise” in the test animals?***

Yes, the rats were removed only after reaching the exhaustion criteria. Due to factors such as whether reagents was administered before exhaustion exercise, differences in administered reagentss, and the rats' individual body weights, there were variations in the duration before reaching exhaustion.

***- The preparation and the amount of myocardial tissue to be prepared for the S1P determination is not mentioned***

The preparation and the amount of myocardial tissue to be prepared for the S1P determination：accurately weigh the weight of animal tissue and add 9 times of the volume of homogenizing medium (0.86% or 0.9% normal saline is recommended) according to the proportion of weight (mg): Volume (UL): 1:9; under the condition of ice water bath, mechanical homogenization is prepared into 10% homogenizing solution, 2500-3000 RPM, centrifugation for 10 minutes, and take the supernatant for determination.

***Results:***

***- The authors refer to tables in several places. However, these cannot be found in the manuscript.***

***- The designation “group C” for the controls is confusing, especially as the figures show “Control”.***

***- Fig. 1A: A reduction in heart weights cannot be seen from the graph alone. Even with the caveat that this is not a significant change, the authors should not describe these heart weights of the groups as different.***

***- When describing and presenting data, one should always compare the treatment with the controls. This alternate presentation (e.g. Results to Fig. 1B) is misleading. Here it would be “were decreased in EP group...”***

We have made correction according to the Reviewer’s comments(P9-10).

*- Fig. 1B:* ***Can the histological effect also be quantified?***

We are very sorry for our negligence of it.

- **Fig. 1D: 75% apoptosis in the myocardium after a single swim seems almost implausibly high to me. Does this match the literature? Is there any way to explain this? The** **animals should hardly be viable after this training.**

Yes,It is really true as Reviewer suggested that the animals should hardly be viable after this training. This why we arranged the rats underwent a single bout of exhaustive swimming exercise.

**- Fig. 2A. It is unclear how this unit comes about. The amount of S1P in the myocardium cannot be given as ng/ml without further information and explanations.**

The pan-species competitive ELISA kit designed for the quantitative detection of Sphingosine-1-Phosphate (S1P). It offers a broad detection range(12.35-1000ng/ml) and high sensitivity(minimum detectable concentration≤ 4.73ng/ml), with a relatively short assay procedure time.

- **Fig. 2A: Are there also differences in plasma S1P?**

Unfortunately, we don't know because we didn't extract rat plasma from the experiment.

**- Fig. 2C: Is there a possibility of quantification? A change in cardiomyocyte rupture, as described by the authors, can hardly be detected.**

Yes, we have made correction.

**- Fig. 2E: Was** **the inhibition of other S1PR also** **tested? S1PR3 in particular also seems to be of interest here**

We are very sorry we don't test the inhibition of other S1PR.

**- Fig. 3A. A sectioned Western blot image is not sufficient to support the statement “the expression of p-MEK in the myocardium of rats... was significantly reduced...”. Quantification with statistically significant differences is required here.**

**- Fig. 3B/C: It is not possible to speak of differences here. The text should be revised.**

*We have made correction according to the Reviewer’s comments.*

**Discussion**

**- The authors do offer a number of references to justify the finding mechanism. However, this is not sufficient. It is possible that the increased formation of S1P via SphK1 also plays a role here. This has already been shown for other cells that were also exposed to a certain amount of stress (Benkhoff et al., Circulation. 2025).**

Recent studies highlight a critical interplay between S1P signaling and the MAPK pathway: endurance exercise upregulates sphingosine kinase 1 (SPHK1) activity, enhancing S1P synthesis. S1P binding to receptors (e.g., S1PR1/S1PR2) activates downstream MAPKs (ERK1/2 and p38), which suppress pro-apoptotic proteins (e.g., Bax), promote mitophagy, and bolster antioxidant defenses; exercise-induced MAPK phosphorylation (e.g., p38) further amplifies SPHK1 expression and activity, establishing a S1P-MAPK positive feedback loop. This axis is particularly robust in slow-twitch myofibers, where mitophagy degrades ceramide (a pro-apoptotic sphingolipid) and elevates S1P levels, optimizing mitochondrial function and endurance adaptation.(P15)

***Reviewer #2: Strengths***

*The experimental design is logical and relevant to the study question.*

*The use of both S1P receptor and MAPK pathway inhibitors helps dissect the mechanisms involved.*

*The figures support the text well and show clear experimental differences.*

*Ethical approval and animal welfare guidelines were followed.*

***Weaknesses and Suggestions for Improvement***

1. ***Sample Size & Randomization: Clarify how rats were randomized into groups. Indicate whether investigators were blinded during analysis.***

We have made correction according to the Reviewer’s comments(4).

2. ***Data Transparency: Include numerical data (mean ± SD) within the main text or supplementary tables. For instance, actual S1P concentrations in each group. Figures are provided, although raw data (e.g., actual ELISA values, full apoptotic counts) could further strengthen transparency.***

***3. Statistics: Indicate whether post hoc tests were used. Also, include effect sizes or confidence intervals where possible. The manuscript lacks a statement on statistical power or sample size calculation. Exact p-values (rather than simply “P < 0.05”) would improve transparency.***

We have made correction according to the Reviewer’s comments.

3. ***Discussion: While generally well-referenced, the discussion would benefit from addressing alternative pathways (e.g., eNOS or STAT3) as mentioned briefly at the end. Consider whether MAPK activation is a downstream result or a parallel pathway influenced by S1P.***

Previous studies have reported that activation of the S1P/S1PR1/STAT3 signaling pathway may promote inflammatory damage and fibrosis of the cardiac valves; and the activation of S1P receptors plays a crucial role in inhibiting the activation of Caspase-3, a key executor of apoptosis, by stimulating the AKT pathway. And the studies suggests that eNOS-knockout mice lose S1P-mediated cardioprotection; endothelial S1PR1 regulates pressure overload-induced cardiac remodelling through AKT-eNOS pathway.(P15)

4. ***Figure Legends: Improve clarity. Example: Figure 2 – instead of “showed increase,” write “was significantly higher than the EP group (P < 0.05).”***

***5. Grammar & Language: Minor editing needed for readability and fluency. A few examples:***

***Replace “cardiomyocyte rupture and interstitial changes were increased” with “cardiomyocyte rupture and interstitial edema were more prominent.”***

***“This study was carried out in accordance with the principles of the Basel Declaration.” → "The study adhered to the Basel Declaration on animal research ethics."***

***"An observable dysregulation of swimming actions..." → Consider simplifying to "evident swimming fatigue."***

***Use active voice more often. Avoid redundant phrases like “compared to the EP group, the EP+W146 group showed…” repetitively.***

***Figures and Tables***

***Figure Quality: Acceptable but can be improved by adding scale bars, clearer labels (e.g., “n = 6/group”), and including actual values in bar charts.***

***Table Clarity: The tables referenced (Table 1–8) ??? are not embedded or visualized in the version reviewed. If these exist separately, ensure they are submitted as part of the supplemental material.***

We have made correction according to the Reviewer’s comments.

**Reviewer #3:** Thank you to the authors for their comprehensive and well-designed study on exercise preconditioning and its effects on myocardial S1P content in rats. The study is timely, and it provides valuable insights into the potential mechanisms underlying exercise-induced myocardial protection. Below are my specific comments aimed at improving clarity, experimental rigor, and presentation of results.

***Introduction***

***Introduction is written summarized and well. The only point that could be mentioned is the relationship of S1P and MAPK in the literature, regardless of preconditioning. Before writing " Previous reports indicate that the MAPK signaling pathway may attenuate cardiac fibrosis, apoptosis, oxidative stress injury, and inflammatory reactions [5]." You can bring a bridge from s1p to MAPK and their importance in preconditioning… .***

Previous reports indicate that endurance exercise may upregulates sphingosine kinase 1 (SPHK1) activity, enhancing S1P synthesis. S1P binding to receptors (e.g., S1PR1/S1PR2) activates downstream MAPKs (ERK1/2 and p38), which suppress pro-apoptotic proteins, promote mitophagy, and bolster antioxidant defenses.(P3)

***Methodological Recommendations:***

***1. Clarification of Exercise Protocol:***

***o The manuscript states that the exercise duration gradually increased from 30 minutes/day to 2 hours/day. It would be helpful to specify how the intensity of the swimming exercise changed during this period. For example, was the swimming intensity (e.g., swim speed or resistance) progressively increased along with the duration? Detailing the intensity would improve the reproducibility of the exercise protocol.***

***2. Control Group and Randomization:***

***o While the animals were randomly assigned to groups, it is essential to explicitly mention whether the experimenters were blinded to the group assignments and the outcome measures (e.g., apoptosis index, S1P levels). Blinding helps reduce potential biases during outcome assessments.***

We have made correction according to the Reviewer’s comments(P3-7).

***3. Sample Size Justification:***

***o While the sample sizes for each group are mentioned (e.g., n=5 or n=6), the manuscript would benefit from a clear justification of how these sample sizes were determined. Were power analyses conducted to ensure that the study had enough statistical power to detect differences between groups? If not, this could be a limitation to address in the revised manuscript.***

The sample size was determined through a power analysis using G*Power software, aiming for 80% power to detect a medium effect size (f² = 0.15) at a significance level of α = 0.05. This calculation ensured that the study had an adequate sample size to detect meaningful differences. Statistical power was considered during the design phase to minimize the risk of Type II errors and ensure the reliability of the results.

***4. S1P Measurement:***

***o The manuscript mentions using an ELISA kit to measure myocardial S1P content. Did you validate the S1P ELISA kit against other methods, such as mass spectrometry, to ensure its accuracy? This validation would strengthen the reliability of the S1P data.***

Because the lower limit of our Mass Spectrometry detection instrument cannot detect the concentration of S1P, an ELISA kit was selected for detection.

5***. Inhibition Timing and Dosing:***

***o The manuscript describes the use of the S1P receptor blocker W146 and MAPK inhibitor PD98059, but the exact timing of administration relative to the exercise sessions could be more clearly defined. Were these inhibitors given before every exercise session or just once a day? Was the dose adjusted over time based on the animals' body weight? These details are critical for reproducibility and understanding the effectiveness of the treatments.***

The exercise duration gradually increased from 30 minutes per day in week 1, 1 hour per day in week2, and 2 hours per day in week 3, conducted 6 days per week. The swimming intensity (e.g., swim speed or resistance) does not progressively increased along with the duration. （P6-7）

6. ***Method of Exhaustion Induction:***

***o The criterion for exhaustion (e.g., observable dysregulation of swimming actions, slow paddling speed, continuous sinking) is subjective. While this is commonly used in exercise studies, addressing this issue in limitation would enhance the clarity of the findings.***

In the study of exercise science, rat swimming experiments are a common method to evaluate fatigue. However, the criteria for judging fatigue, such as swimming movement disorders, slow paddling speed, and continuous sinking, do indeed have a certain degree of subjectivity.

Results and Data Presentation:

***1. Figures***

***o It would be informative to provide the exact data or statistical analysis in the figure legend to allow the reader to understand how significant the difference is between groups.***

We have made correction according to the Reviewer’s comments.

o ***In Figure 3E, apoptosis is increased in the EP + PD98059 group. A discussion about the mechanisms contributing to this finding (i.e., how MAPK inhibition could increase myocardial damage despite exercise preconditioning) would be helpful to provide a more comprehensive interpretation of the data.***

Studies show that PD98059 exacerbates myocardial injury in exercise-precondition by blocking survival signals, inhibiting endogenous protectants and aggravating oxidative stress, underscoring the indispensable role of MAPK in adaptive cardioprotection,which can explain why apoptosis increases

***Reviewer #4: Comment 1: The tables are not visible in the main text. Please include them at the end of the manuscript instead of submitting them as a supplementary file.***

We have made correction according to the Reviewer’s comments.

***Comment 2: While the study suggests MAPK signaling is involved in S1P-mediated protection, the molecular mechanism remains speculative. Consider discussing alternative or complementary signaling pathways such as AKT or STAT3, which were briefly mentioned but not tested.***

Previous studies have reported that activation of the S1P/S1PR1/STAT3 signaling pathway may promote inflammatory damage and fibrosis of the cardiac valves[21]; and the activation of S1P receptors plays a crucial role in inhibiting the activation of Caspase-3, a key executor of apoptosis, by stimulating the AKT pathway[22]. And the studies suggests that eNOS-knockout mice lose S1P-mediated cardioprotection[23]; endothelial S1PR1 regulates pressure overload-induced cardiac remodelling through AKT-eNOS pathway.(P15)
