# Supplementary material for: Effect of exercise preconditioning on myocardial content of Sphingosine1-phosphate and its mechanism in rats after exhaustive exercise
Source: PLoS One. 2026 Jan 7;21(1):e0340313. doi: 10.1371/journal.pone.0340313 (PMC12779141; doi:10.1371/journal.pone.0340313)
Supplement: Attachment — Submitted filename: Response to Reviewers_updated.doc [file pone.0340313.s008.doc]

**Dear Dr. Amirreza Khalaji and Reviewers,**

Thank you for your letter and the opportunity to revise our manuscript, “Effect of Exercise Preconditioning on Myocardial Content of S1P and its Mechanism in Rats after Exhaustive Exercise” (ID: PONE-D-25-14601R1). We sincerely appreciate the reviewers’ insightful comments, which have greatly helped us improve the quality of our work.

We are pleased to note that most reviewers found the revised version acceptable. In this point-by-point response, we have addressed all remaining comments thoroughly, with particular attention to the concerns raised by Reviewer #5. To facilitate your review, all revisions have been made using the **'Track Changes' feature**; deletions and additions are clearly marked in the text.

Below, we provide our detailed responses to each comment.

Thank you for your letter and the opportunity to revise our manuscript, "Effect of Exercise Preconditioning on Myocardial Content of S1P and its Mechanism in Rats after Exhaustive Exercise" (ID: ). We are sincerely grateful for the constructive feedback throughout the review process, which has greatly strengthened our paper.

**We are delighted to note that Reviewers #3, #6, and #7 have found all their previous concerns adequately addressed and consider the manuscript acceptable for publication. We extend our sincere thanks for their time and valuable input.**

In this point-by-point response, we have thoroughly addressed the comments from Reviewer #5 and Reviewer #8. All corresponding changes in the manuscript have been highlighted for your convenience.

Below are our detailed responses to the comments from Reviewers #5 and #8.

**Reviewer #5**:

**The revision does not provide proper, point-by-point answers to the reviewers’ comments; many substantive issues remain unaddressed. For example, a required Declarations section is still missing (ethics approval/consent, data availability with an accessible link, funding, competing interests, and author contributions).**

Thank you for your feedback and for bringing this to our attention. We sincerely apologize for the oversight in our previous submission. The point-by-point response to the reviewers' comments was inadvertently omitted during the upload process. We have now uploaded the "Response to Reviewers" document to the submission portal. This document provides a detailed, point-by-point account of all changes made in response to the reviewers' valuable comments. Regarding the Declarations section, we confirm that all required statements, including the **ethics approval and consent which are detailed in section 1.2.4 "Ethics Statement" of the manuscript**, are fully included. The complete Data Availability statement with the accessible link is also present within the main manuscript file.

We regret any confusion or inconvenience this omission may have caused. Thank you for your patience and the opportunity to correct this error.

**The manuscript investigates whether exercise preconditioning (EP) mitigates exhaustive-exercise–induced myocardial injury in rats and explores a mechanistic pathway involving sphingosine-1-phosphate (S1P) and MAPK signaling across four groups (Control, EP, EP+W146, EP+PD98059), with outcomes including heart weight, histology, apoptosis, S1P levels, and p-MEK.** **This is a revised submission; however, substantive issues remain in methodological reporting (e.g., blinding, euthanasia parameters, randomization, sample-size justification), statistical transparency (tests, assumptions, multiple-comparison control, effect sizes/CIs), and internal consistency between text, tables, and figure legends. Several contradictions (e.g., non-significant results described as decreases) and formatting/artifact problems persist, affecting interpretability and reproducibility. In its current form, the study requires major revision to meet PLOS ONE’s rigor and reporting standards.**

Major comments

1. **Results describe a decrease “not statistically significant,” and the table shows no difference (e.g., p≈0.95), yet the Figure 3 legend asserts a decrease. All sections must agree and use neutral wording that reflects the statistics.**

**Response:** We sincerely apologize for the inaccurate description of our results. The legend of Figure 3 has been corrected to accurately reflect the statistical findings. A comprehensive review of the entire manuscript has been conducted to ensure internal consistency, and we have revised the relevant text to clearly convey the non-significant changes observed in the data. We greatly appreciate your meticulous and constructive feedback.

2. **Results indicate no significant difference (P>0.05), but the figure legend states “Cardiac weight was increased.” Legend phrasing should mirror the statistical outcome.**

**Response:** Thank you for bringing this inconsistency to our attention. Upon careful review, we realize that there was an error in the figure 3C legend. The legend should indeed reflect the statistical outcome, which is that there was no significant difference in cardiac weight between the EP group and EP + PD98059 group.We apologize for this oversight and have updated the legend to accurately reflect the results. The revised legend now reads: "...showed no significant difference between the EP and EP+PD98059 groups (P = 0.849, Cohen's d = 0.12, 95% CI [-1.25, 1.49])” (**on page27-28, fig 3**). We appreciate your thorough review and are committed to ensuring the accuracy and clarity of our manuscript. Please let us know if there are any further concerns or if you need any additional information. Thank you again for your assistance in improving our paper.

3. **Table content contains a merged/duplicated line and mixes mean±SD with median(IQR) for the same endpoint. Clean the table and use one summary metric aligned with the applied test.**

**Response:** We are very sorry for our incorrect writing in mixing the use of mean ± standard deviation and median (interquartile range). Tables have been revised to remove the duplicated line. Continuous variables with normal distribution were uniformly presented as mean ± standard deviation (SD). All values are now standardized, and for clarity, the table has been cleaned. These updates ensure accuracy and alignment with statistical methods. Thank you for the valuable feedback.

4. **CO₂ flow is reported as “3-70% chamber volume per minute,” and elsewhere** **euthanasia is described under deep anesthesia “when necessary.”** **Specify one guideline-concordant method with exact parameters and apply it consistently.**

**Response:** We thank the Reviewer for the feedback. We have updated the manuscript to specify a single CO2 inhalation euthanasia protocol per AVMA guidelines, with full details in Methods and supplementary materials. All procedures followed IACUC-approved protocols, enhancing transparency, reproducibility, and ethical rigor.

5. **Blinding is stated despite visibly different procedures. Specify who was blinded, how (coding/masking), and which outcomes were assessed blinded (e.g., coded histology/apoptosis); otherwise revise to** **assessor-blind.**

**Response:** Due to the visibly different procedures, complete blinding was not feasible.We have state “To minimize bias in data acquisition and analysis, **an assessor-blinded study design was implemented throughout the experimental procedures**. Personnel responsible for conducting biochemical assays (ELISA, Western blot), performing histopathological evaluations, and scoring TUNEL assays were unaware of the group assignments until all quantitative analyses were completed”.(**on page9-10, 1.6 Blind Analysis Protocol**).

6. **P****ower is reported using a regression effect size (f²=0.15) for a four-group design; achieved n≈5–6/group is small for histology/apoptosis. Provide an ANOVA/GLM-appropriate calculation (primary outcome, effect size, α, power) or state that the study is exploratory.**

**Response:** We thank the reviewer for raising this important point regarding statistical power. In response, we have revised the manuscript to explicitly frame our study as exploratory in nature. As detailed in the Methods section, we state that “the sample size of n=5-6 per group was determined for this exploratory study...”(**on page 6, 1.3 Experimental design, grouping, and animal models**). We believe this clarification accurately positions our work and sets appropriate expectations for the reader.

We believe this framing accurately sets reader expectations and strengthens the transparency of our work.

7. **P-values are reported without naming the tests, assumption checks, multiple-comparison handling, effect sizes, or 95% CIs. Add a Statistics subsection with software, tests/models per endpoint, diagnostics, and corrections; report estimates with CIs.**

**Response:** We sincerely thank the reviewer for this critical and constructive comment regarding the reporting of our statistical analyses. We fully agree that transparent and comprehensive reporting is essential for reproducibility and rigor. In response, we have thoroughly revised the manuscript as follows:

(1). **Added a Dedicated 'Statistical Analysis' Subsection**: A new "Statistical Analysis" subsection has been added to the Methods. It explicitly states:

-The statistical software used (SPSS version 23.0 and GraphPad Prism version 10.0).

-The specific test applied for each comparison (e.g., Student's t-test, one-way ANOVA).

-How assumptions were checked, including normality (Shapiro-Wilk test) and homogeneity of variances (Levene's test).

-That post-hoc comparisons following significant ANOVA results were performed using Tukey's Honest Significant Difference (HSD) test to correct for multiple comparisons.

(2) . **Enhanced Reporting of Statistical Results:**

Throughout the Results section, figure legends, and tables, P-values are now accompanied by the name of the test used. Furthermore, effect sizes (e.g., Cohen's d) and 95% confidence intervals (CIs) have been included where appropriate, providing a more complete and interpretable statistical summary.

We believe these comprehensive revisions have significantly strengthened the statistical rigor, transparency, and reproducibility of our manuscript. We are grateful for the reviewer's insightful suggestion, which has greatly improved the quality of our work.

8. **Clarify whether heart weight is absolute or normalized (and how), define apoptosis index quantification (fields, thresholds, blinding), provide S1P kit/catalog details, and include n/group in figure panels.**

**Response:** We thank the reviewer for these important requests for clarification. We have revised the manuscript accordingly to ensure full transparency, and the specific details are as follows:

(1). **Heart Weight**: We confirm that all heart weight data are presented as absolute values (in grams).As detailed in the Methods section we state that “...**the hearts were rapidly excised, weighed**, and rinsed with sterile saline”**(on page 7-8, 1.4 Euthanasia and tissue collection**).

(2). **Apoptosis Index Quantification**: The quantification method for the TUNEL assay has been detailed in the Methods. Briefly, TUNEL-positive cardiomyocytes were counted in three random fields (200× magnification) per section. Positive cells were identified based on predefined morphological criteria (e.g., pyknotic nuclei) and distinct, intense nuclear staining. All counts were performed by an investigator blinded to the group assignments**(on page 9, 1.5.3 TUNEL assay)** .

(3). **S1P ELISA Kit**: The full details of the commercial ELISA kit used for S1P measurement, including the supplier (Wuhan Yunclone, China) and the catalog number (UCEG013Ge), have been added**(on page 3, 1.1 Primary Reagents and Kits**).

(4). **Sample Size in Figures:** The sample size (n) for each experimental group is now clearly indicated in all figure panels and legends (e.g., n=5 per group). All representative images include scale bars.

We believe these revisions have significantly enhanced the methodological clarity and reproducibility of our study.

9. **Randomization is stated without method/timing (sequence generation, concealment, independent allocator). Provide concrete implementation steps.**

**Response:**We thank the reviewer for the insightful comment. In our rat study, randomization was rigorously implemented.The random sequence was generated using computer-generated block randomization (block size 4-6) via SAS 9.4, stratified by body weight and baseline measurements. Allocation concealment was ensured using sequentially numbered, sealed, opaque envelopes prepared by a statistician not involved in the experiment. Envelopes were opened only after the rat was enrolled and baseline data recorded. An independent researcher, unrelated to animal housing or treatment, performed the allocation. Randomization occurred immediately before intervention initiation. These steps ensured unbiased group assignment, with full transparency and reproducibility. The methodology has now been clarified in the Methods section (**on page 6, 1.3 Experimental design, grouping, and animal models**).

Minor comments

10. **Figure legends—units and n/group: Add units (e.g., S1P ng/mL, °C) and n/group in each legend; ensure legend wording reflects the actual statistical outcome (e.g., “no significant difference” where applicable).**

**Response:**We thank the reviewer for the valuable feedback. We have revised all figure legends to include clear units, specify n/group , and accurately describe statistical outcomes. These updates enhance clarity, consistency, and alignment with the data and analysis, improving the overall rigor of the figures.

11. **Data availability / ethics alignment. Ensure the Data Availability and Ethics statements match Methods with precise institutional approval details and access to raw per-animal data (and original images where applicable).**

**Response:**We thank the reviewer for highlighting the importance of data transparency and precise ethical reporting.

**(1). Data Availability**: As required by PLOS ONE policy, all raw per-animal data supporting the findings of this study are available in the Figshare repository at [<https://figshare.com/s/e4f4442f0cbdf3c3f557>]. The detailed experimental protocol is available on Protocols.io at [https://www.protocols.io/private/027003F9B0D911F082020A58A9FEAC02].

**(2).Ethics Statement**: We have thoroughly revised the Ethics Statement in the Methods section to provide precise and unambiguous details. The updated statement now includes:

-The full and correct approval number, prefixed with "IACUC-" for clear identification.

-A standardized date format.

-Explicit reference to the specific international guidelines followed-the ARRIVE guidelines 2.0 and the NIH Guide for the Care and Use of Laboratory Animals-ensuring our research aligns with the highest international standards.

**The updated text now reads**:

“1.2.4 Ethics Statement: All animal experiments were approved by the Institutional Animal Care and Use Committee (IACUC) of NingDe Municipal Hospital (Approval No.: IACUC-20200310) on March 21, 2020. All procedures were performed in strict accordance with the National Standard of China (GB/T 35892-2018) and followed the ARRIVE guidelines 2.0 as well as the U.S. National Institutes of Health Guide for the Care and Use of Laboratory Animals”**(on page 6, 1.2.4 Ethics Statement)**.

We believe these revisions fully address the reviewer's concerns.

**Reviewer #8:**

**-I checked all revision files, unfortunately I can't find point by point response letter or section from authors.**

Please accept our sincere apologies for this oversight. The point-by-point response letter was inadvertently omitted. We have now uploaded the file "**Response to Reviewers_updated.doc**" to the submission portal.

Thank you for your patience and for allowing us to correct this error.

**Dear Authors**

**Thank you for your submission and for the substantial effort invested in a valuable. Yor revision sounds good and almost complete, but there are minor things to care. The comments that follow are offered in a constructive spirit to help sharpen the manuscript’s clarity and maximize its usefulness to researchers.**

My comments:

-**Reconcile the direction of S1P change (EP vs Control) across text, tables, and figures; ensure the narrative matches the numeric values and final conclusions.**

**Response:**Thank you for your insightful comment. We have corrected inconsistencies in the S1P change (EP vs. Control) across text, tables, and figures, aligning the narrative with the data and conclusions. We appreciate your guidance in enhancing our manuscript’s accuracy.

**-****Consolidate to one Statistics section (remove legacy one-way ANOVA text if not used).**

**Response:**Thank you for the valuable comment. We have consolidated all statistical descriptions into a single "Statistical Analysis" section. The one-way ANOVA was indeed used to analyze the Western blot densitometry data and has been clearly included in the revised section**(on page 27, Fig 3 legend).**

**-****Name a single primary endpoint (used for power) and map each outcome (heart weight, S1P, apoptosis, p-MEK) to: -distribution check (e.g., Shapiro–Wilk),-summary measure (mean±SD or median[IQR]), exact test used, post-hoc method for the 4-group design, multiple-comparison control (e.g., Tukey, Dunn, Holm/Benjamini–Hochberg), effect sizes and 95% CIs.**

**Response:** We are deeply grateful for your rigorous and constructive comments regarding the statistical analysis in our manuscript. We sincerely apologize for the initial lack of detail. We have consulted with a biostatistician to ensure the accuracy of our approach and have thoroughly revised the 'Statistical Analysis' section in the Methods, as well as the results presentation, to incorporate all the points you raised.

Below is a point-by-point response to your specific requests.

**1. Single Primary Endpoint (for power):**

As recommended, we have designated the S1P level as the single primary endpoint for our study(**on page 10, 1.7 Statistical Analysis**).

**2. Sample Size Justification:**

The sample size of n=5-6 per group was determined for this exploratory study. As the primary goal was to investigate novel mechanistic insights into the S1P/MAPK pathway and identify robust effect trends for generating future hypotheses, a formal a priori power calculation was not performed. The sample size was selected based on common practices in preliminary physiological research and practical feasibility. To facilitate interpretation, we have prioritized the reporting of effect sizes with 95% confidence intervals for all key outcomes(**on page 6, 1.3 Experimental design, grouping, and animal models**).

**3. Statistical Mapping for Each Outcome:**

We have now explicitly mapped the statistical approach for each outcome as requested. The table below details the specific statistical tests that were actually applied to each outcome measure, based on the results of the normality and homogeneity of variance tests.

| Outcome Measure | Distribution Check | Summary Measure | Exact Test Used | Post-hoc & Multiple Comparison Control | Effect Size & 95% CI |
| --- | --- | --- | --- | --- | --- |
| ****S1P level**** (Primary) | Shapiro-Wilk | Mean ± SD | **Independent t-test** | Not Applicable | **Cohen's d**; 95% CI |
| ****Myocardial apoptosis rate**** | Shapiro-Wilk | Mean ± SD | **Independent t-test** | Not Applicable | **Cohen's d**; 95% CI |
| ****Heart weight**** | Shapiro-Wilk | Mean ± SD | **Independent t-test** | Not Applicable | **Cohen's d**; 95% CI |
| ****p-MEK level**** | Shapiro-Wilk | Mean ± SD | One-way ANOVA | **Tukey's HSD** | η²; 95% CI |

**-****Resolve software(version) inconsistencies (e.g., SPSS 17 vs newer; Prism) and state the final versions only.**

**Response:** We apologize for the inconsistency in software versions previously reported in the manuscript. In response to the reviewers’ comments, we have fully standardized the software platforms used in this study: all statistical analyses were conducted using **IBM SPSS Statistics version 23.0**, and all graphs and data visualizations were generated with **GraphPad Prism version 10.0**. All results and figures have been re-verified using these specified versions to ensure methodological consistency, transparency, and reproducibility. Furthermore, we have carefully reviewed the entire manuscript and updated all references to software versions accordingly.

**-****Use W146 = S1PR1-selective antagonist; PD98059 =** **MEK1/2 (MAPKK) inhibitor.**

**Remove any remaining “MAPK blocker” wording in text, tables, and figure legends.**

**Response:** We thank the reviewer for this precise and helpful comment. We have revised the manuscript accordingly. Specifically:

(1). We have replaced all instances of non-specific terms with the precise nomenclature as suggested:

-“W146” is now defined as “S1PR1-selective antagonist” throughout the text, tables, and figure legends.

-“PD98059” is now defined as “MEK1/2 (MAPKK) inhibitor” throughout the text, tables, and figure legends.

(2). We have carefully removed any remaining “MAPK blocker” wording from the entire manuscript, including the main text, tables, and all figure legends.

These changes have been incorporated into the revised manuscript. We believe these modifications have significantly improved the precision and clarity of our work.

**-Report myocardial S1P normalized to tissue mass (ng/g) or protein (ng/mg); if retaining ng/mL homogenate, provide a clear calculation pipeline (homogenization volumes, protein assay) so others can reproduce it.**

**Response:** We thank the reviewer for this valuable suggestion. We have revised the units for myocardial S1P from "ng/mL homogenate" to "ng/mg protein" as recommended.

To ensure clarity and reproducibility, we have added the following detailed calculation pipeline to the Methods section:

(1). **Homogenate Preparation**: Precisely weighed myocardial tissue (≈100 mg) was homogenized in 1 mL of ice-cold PBS.

(2). **Protein Assay**: The total protein concentration of the homogenate was determined using a BCA assay.

(3). **S1P Quantification & Normalization:** S1P levels were measured in the homogenate by ELISA. The final concentration was normalized to tissue protein content using the formula:

[S1P] (ng/mg protein) = [S1P] in homogenate (ng/mL) / Protein concentration (mg/mL)

For illustrative purposes:

*If [S1P] in homogenate = 8.0 ng/mL and Protein concentration = 10.0 mg/mL, then the reported value = 8.0 / 10.0 = 0.8 ng/mg protein.*

The description in the manuscript is correct: the final S1P concentrations were indeed normalized to the total protein content of the myocardial tissue homogenate, and the results are expressed as nanograms of S1P per milligram of total protein (ng/mg protein)**( on page 8, 1.5.1 Quantification of myocardial S1P)**.

We believe this revision fully addresses the reviewer's concern.

**-Add operational details, who generated or kept the randomization list, how allocation was concealed, who assigned animals, who was blinded for TUNEL scoring and Western densitometry, and when blinding was lifted.**

**Response:**We thank the reviewer for raising this important point. We have now revised the 'Methods' section to incorporate all requested operational details. The changes are highlighted in the manuscript. Specifically:

1. **Randomization List**: The random sequence was generated using computer-generated block randomization via SAS 9.4 by a statistician not involved in the experiment. **Allocation Concealment**: This was ensured using sequentially numbered, sealed, opaque envelopes prepared by the same statistician. **Animal Assignment**: An independent researcher, unrelated to animal housing or treatment, performed the allocation by opening the envelopes after rat enrollment.(**on page 6, 1.3 Experimental design, grouping, and animal models**)

**2.Blinded Personnel**: To minimize bias in data acquisition and analysis, an assessor-blinded study design was implemented throughout the experimental procedures. Personnel responsible for conducting biochemical assays (ELISA, Western blot), performing histopathological evaluations, and scoring TUNEL assays were unaware of the group assignments until all quantitative analyses were completed.**(on page 9-10,1.6 Blind Analysis Protocol)**

**Lifting of Blinding: The blinding was maintained until all quantitative analyses were completed.**

**-Reconcile inconsistent euthanasia, and anesthesia descriptions; ensure the Methods reflect the actual procedure and are consistent across manuscript and submission materials.**

**Response:** We sincerely thank the reviewer for highlighting the inconsistency in the description of the euthanasia procedure. We apologize for this oversight. We have now thoroughly revised the manuscript to ensure a completely consistent and accurate account across all sections.

The detailed, step-by-step protocol originally described in section 1.2.2 represents the full and correct procedure that was applied to all animals. To eliminate redundancy and potential confusion, we have revised the brief mention in section 1.4 to directly reference the detailed description. The text in 1.4 now reads:

"Following euthanasia, which was performed thirty minutes post-modeling as detailed in section 1.2.2..."(**on page7, 1.4 Euthanasia and tissue collection**).

We confirm that the methodology is now consistent throughout the manuscript and accurately reflects the actual procedure performed.

**-Include representative images with scale bars.**

**Response:** We deeply regret the inadvertent omission of the scale bars in Fig. 1B and Fig. 3E, and have promptly rectified this oversight by incorporating them into the revised figures. We appreciate the opportunity to address this issue and ensure the integrity and clarity of our presentation.

**-****Describe field selection, counting thresholds, automation, and positive-negative controls to address potential TUNEL over-calling.**

**Response:**Thank you for this important question. To minimize TUNEL over-calling, we implemented a rigorous protocol**(on page 9, 1.5.3 TUNEL assay)**:

**1.Blinded, Systematic Sampling**: Fields were selected systematically at 200x magnification from three random sections per animal by a blinded observer.

**2.Automated, Threshold-Based Quantification**: Apoptotic index (TUNEL+ nuclei / total nuclei) was quantified using automated image analysis. Positive signals were defined by stringent, pre-validated thresholds for nuclear size and intensity.

**3.Specificity Controls**: Each batch included DNase-treated (positive) and enzyme-omitted (negative) controls to confirm assay specificity.

**4.Blinded Verification**: All automated counts were independently verified by two blinded observers, with discrepancies resolved by consensus.

**-****Remove tracked-changes artifacts, duplications; correct any duplicated or corrupted table entries.**

**-Ensure each legend lists n/group, exact test, post-hoc method, and exact P-value criteria.**

**Response:** We have thoroughly revised the manuscript to address the reviewer's formatting concerns. All tracked-changes artifacts and duplications have been removed, table entries have been verified and corrected, and all figure legends now clearly specify the n per group, exact statistical test, post-hoc method, and P-value criteria.

**-Specify exact timing of W146 and PD98059 (relative to each preconditioning session and to the exhaustive swim), vehicle, dose per kg, and whether dosing changed over time.**

**Response:** We have re-written this part according to the Reviewer’s suggestion, the details as follows: The EP + W146 group received an intraperitoneal injection of W146 (20 mg/kg) 10 min prior to the commencement of each exercise session. The EP + PD98059 group also received PD98059 (100 mg/kg) via identical timing and route (i.p., 10 min before exercise). All inhibitors were given before every exercise session, with doses adjusted according to body weight(**on page 7, 1.3 Experimental design, grouping, and animal models)**.

**-State clearly what is novel (S1P→MAPK inhibition within an exercise-preconditioning model) versus confirmatory, and situate findings against the most relevant prior EP/S1P cardioprotection literature.**

**Response:**We sincerely thank the reviewer for this critical comment, which has helped us refine the narrative of our exploratory study. We have revised the Discussion to more clearly distinguish between confirmatory observations and potential novel insights, and to better situate our findings within the existing literature.

**1.** **The Confirmatory Aspect: Consistency with Established Knowledge**

Our findings align with and reinforce two well-established concepts in the field of exercise cardioprotection:

(1).The general cardioprotective effect of exercise preconditioning (EP), as demonstrated by the significant attenuation of myocardial injury in the EP group (Fig. 1).

(2).In this exploratory study, our findings suggest that S1P may serve as a key mediator in exercise-induced cardioprotection. We observed that exercise preconditioning (EP) elevates myocardial S1P, and that S1PR1 antagonism diminishes this protective effect (Fig. 2). These results build upon the correlation established by Baranowski et al. ^[20]^—between exercise and increased S1P levels—by pointing to a potential causal and protective mechanism in the heart.

**2. The Novel Hypothetical Contribution: A Proposed Hierarchical Signaling Axis**

The potential novelty of our work lies in moving beyond established correlations to propose a testable hierarchical relationship between S1P and MAPK signaling within the specific context of our EP model.

While recent literature, such as the work by Leng et al. ^[5]^, elegantly describes a complex S1P-MAPK feedback loop in skeletal muscle adaptation, our data provide preliminary yet directed evidence for a predominant, unidirectional signaling flow (S1P → MAPK) in exercise-induced myocardial protection. The critical evidence supporting this interpretation comes from our EP+PD98059 group (Fig. 3), where MAPK inhibition abrogated the protective effects of EP but did not alter the elevated S1P levels. This pattern suggests that MAPK activation is indispensable for protection and likely functions downstream of S1P in this paradigm.

Therefore, we propose the S1P→MAPK axis not as a definitive pathway, but as a plausible and parsimonious mechanistic model that best explains our complete dataset. This represents a novel conceptual framework for understanding EP-mediated protection, explicitly presenting a focused hypothesis for future confirmatory research aimed at validating this specific signaling hierarchy.

**SUGGESTIONS FROM THE EDITOR**

**Comment:** If applicable, we recommend that you deposit your laboratory protocols in protocols.io to enhance the reproducibility of your results. Protocols.io assigns your protocol its own identifier (DOI) so that it can be cited independently in the future. For instructions see: https://journals.plos.org/plosone/s/submission-guidelines#loc-laboratory-protocols. Additionally, PLOS ONE offers an option for publishing peer-reviewed Lab Protocol articles, which describe protocols hosted on protocols.io. Read more information on sharing protocols at: https://plos.org/protocols?utm_medium=editorial-email&utm_source=authorletters&utm_campaign=protocols.

**Response:**Thank you for this recommendation. We agree that enhancing the reproducibility of our work is of paramount importance.

In accordance with PLOS ONE's guidelines, we have now deposited the detailed, step-by-step laboratory protocol for the key methodologies used in this study on protocols.io. Furthermore, all supporting raw data and original images have been publicly deposited in the Figshare repository.

The access information for these resources has been integrated into the Data Availability Statement within the revised manuscript, which now reads:

Data Availability Statement: All relevant data, including raw per-animal data and original images, are available from the Figshare repository (DOI: [<https://figshare.com/s/e4f4442f0cbdf3c3f557>]). The detailed experimental protocol is available from the protocols.io repository (DOI: [https://www.protocols.io/private/027003F9B0D911F082020A58A9FEAC02]).

We believe these measures will fully facilitate the verification and replication of our findings.

We look forward to receiving your final assessment of our revised manuscript.

Sincerely,

[Weiyuan Yang]

[Department of Rehabilitation Medicine, Ningde Municipal Hospital of Ningde Normal University, Ningde Fujian, P.R. China]

[yangwy201509@163.com]
